# Chromatin-Bound Cullin-Ring Ligases: Regulatory Roles in DNA Replication and Potential Targeting for Cancer Therapy

**DOI:** 10.3389/fmolb.2018.00019

**Published:** 2018-03-13

**Authors:** Sang-Min Jang, Christophe E. Redon, Mirit I. Aladjem

**Affiliations:** Developmental Therapeutics Branch, Center for Cancer Research, National Cancer Institute, Bethesda, MD, United States

**Keywords:** DNA replication, chromatin, ubiquitin ligases, cancer, therapy

## Abstract

Cullin-RING (Really Interesting New Gene) E3 ubiquitin ligases (CRLs), the largest family of E3 ubiquitin ligases, are functional multi-subunit complexes including substrate receptors, adaptors, cullin scaffolds, and RING-box proteins. CRLs are responsible for ubiquitination of ~20% of cellular proteins and are involved in diverse biological processes including cell cycle progression, genome stability, and oncogenesis. Not surprisingly, cullins are deregulated in many diseases and instances of cancer. Recent studies have highlighted the importance of CRL-mediated ubiquitination in the regulation of DNA replication/repair, including specific roles in chromatin assembly and disassembly of the replication machinery. The development of novel therapeutics targeting the CRLs that regulate the replication machinery and chromatin in cancer is now an attractive therapeutic strategy. In this review, we summarize the structure and assembly of CRLs and outline their cellular functions and their diverse roles in cancer, emphasizing the regulatory functions of nuclear CRLs in modulating the DNA replication machinery. Finally, we discuss the current strategies for targeting CRLs against cancer in the clinic.

## Structure and regulation of CRLs

**Figure 1:**
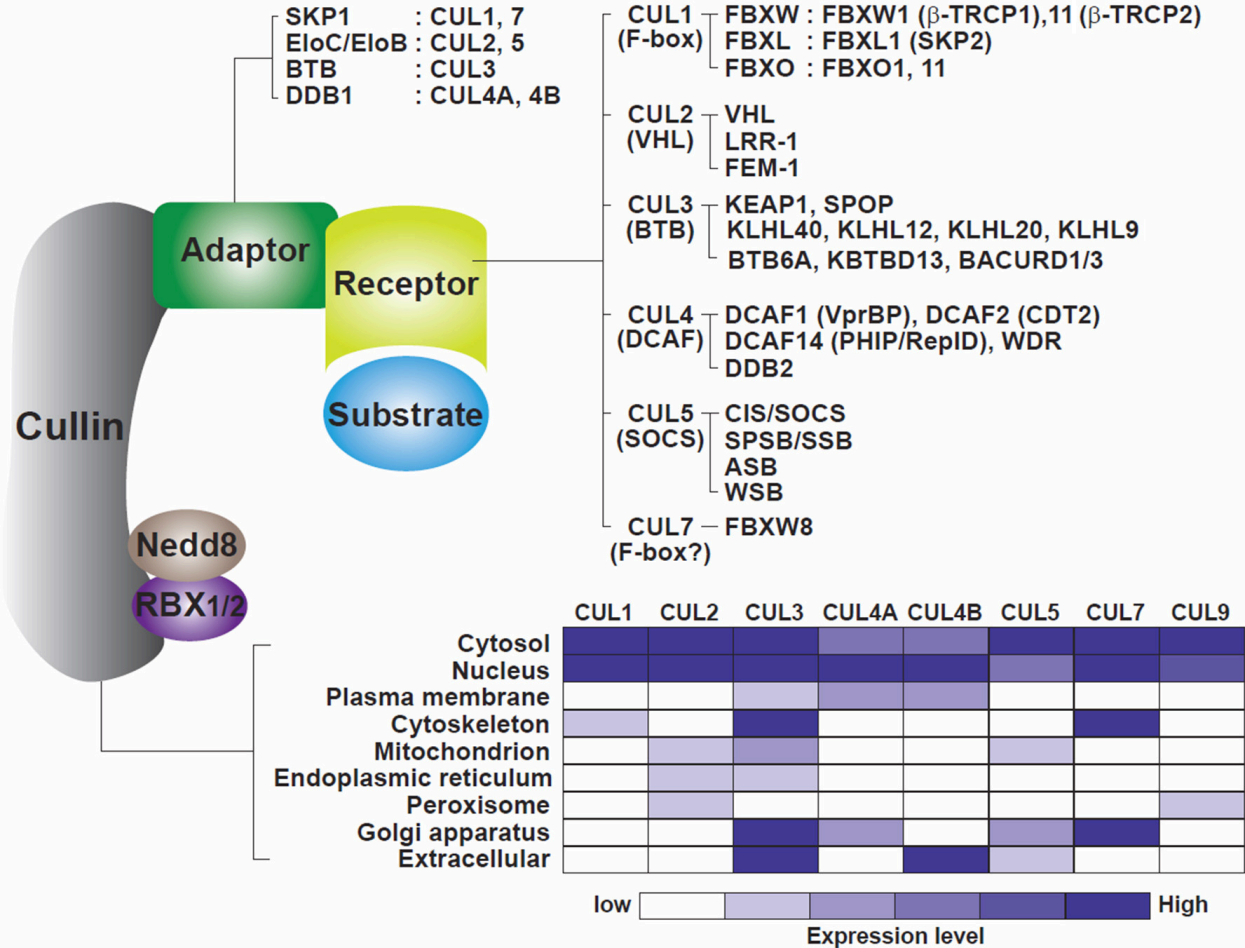
Model of the cullin-RING ligase complexes. Cullins 1, 2, 3, 4A, 4B, 5, 7, and 9 are scaffold proteins that assemble with RING finger proteins (RBX1/2), adaptor proteins (SKP1, EloC/EloB, BTB, DDB1) and receptor or substrate recognition proteins (F-box family, VHL family, BTB, DCAF family, SOCS family among others). A non-exhaustive but known list of the CRLs, adaptors, receptors, and RING proteins is shown. The bottom part of the figure illustrates the cellular localization of the cullins. Cullins are broadly distributed in the different compartments of the cells with CUL4A and CUL4B mostly located in nuclei (table constructed from data gathered from Genecards.org).

CRLs are composed of four components (Figure 1): cullins as molecular scaffolds, adaptor proteins, substrate receptors at the N-termini of cullins, and RING proteins at the C-termini of cullins, recruiting ubiquitin-loaded E2 enzymes (Bulatov and Ciulli, 2015). The evolutionarily conserved cullin family encompasses eight key members (CUL1, 2, 3, 4A, 4B, 5, 7, and 9) that exhibit similar structural architectures and contain cullin homology domains (Sarikas et al., 2011). Activation of CRLs is commonly regulated by NEDD8 modifications at lysine residues located at the C-termini of cullins (Soucy et al., 2010). Otherwise, individual CRLs include specific components, employing substrate receptors as critical determinants of substrate specificity.

CRL1, also known as SCF (SKP1-Cullin 1-F box protein), utilizes S-phase kinase-associated protein 1 (SKP1) as an adaptor protein and recognizes its substrates through substrate recognition proteins known as F-box proteins, which contain 40-amino-acid F-box domains (Zheng et al., 2002). Sixty nine F-box proteins are known to be encoded by the human genome to date and are classified into sub-groups based on their different substrate binding domains, including FBXW (F-box and WD40 domains) FBXL (F-box and leucine-rich repeats) and FBXO (F-box only) (Skaar et al., 2013). CRL2 and CRL5 share an identical adaptor, Elongin C (EloC), known to enhance the rate of RNA polymerase II elongation (Bradsher et al., 1993), and utilize either von Hippel-Lindau (VHL) or suppressors of cytokine signaling (SOCS)-box proteins as distinct substrate receptors (Muniz et al., 2013; Cardote et al., 2017). CRL3 interacts with several BTB (Bric-a-brac, Tramtrack, Broad-complex) domain-containing proteins that implement dual functions as adaptor and receptor subunits (Pintard et al., 2004). The BTB domains of these subunits act as adaptors by associating with CUL3 and RBX1, whereas their MATH (meprin and TRAF homology) motifs and Kelch beta-propeller repeat and zinc finger motifs recognize the substrates (Stogios et al., 2005). CRL4 is anchored by two highly similar scaffold proteins, CUL4A and CUL4B, and an adapter, DDB1 (damage-specific DNA binding protein 1). DDB1 contains three WD40 propeller domains (BPA, BPB, and BPC) and links the CUL4 scaffold with multiple substrate receptors termed DCAFs (DDB1-CUL4-associated factors). Over 100 DCAFs have been identified to date (Zimmerman et al., 2010; Harper and Tan, 2012). CRL7 and CRL9 contain the two largest cullin scaffold proteins, CUL7 (1698 amino acids) and CUL9 (2517 amino acids). As these two cullins are much larger than the cullins anchoring the other CRLs (745–913 amino acids), CRL7/9 may have additional specific, unique functions and/or protein partners. CRL7 is similar to CRL1 in that it includes SKP1 as an adaptor and FBXW8 as a substrate receptor (Dias et al., 2002; Sarikas et al., 2008), but unlike CUL1, it does not interact with the adaptor/receptor complexes SKP1/βTRCP2 or SKP1/SKP2 (Dias et al., 2002). These variations delineate distinct ubiquitin-dependent proteolysis pathways that may be involved in the degradation of specific substrates involved in specific cellular processes and /or in specific cell compartments. Cellular activities of the different CRLs can be co-regulated. For example, CUL7 (with OBSL1 and CCDC8) regulates CUL9 and its substrates to maintain genome stability (reviewed in Jackson, 2014) while degradation of the CRL4 component CDT2 can be orchestrated by the CRL1 (CUL1/FBXO11) complex (Abbas et al., 2013; Abbas and Dutta, 2017).

CRLs are involved in diverse biological processes including cell cycle control, DNA replication, DNA-damage repair, and chromatin remodeling through the selective degradation of various protein substrates, mediated by specific interactions with various substrate receptors. As an example, the SCF (CRL1) complex plays a vital regulatory role by ubiquitinating a series of cell cycle regulators including EMI1 (Margottin-Goguet et al., 2003), CDC25A and B (Busino et al., 2003; Kanemori et al., 2005), WEE1A (Watanabe et al., 2004), Cyclin D1 (Wei et al., 2008), and PTTG1/Securin (Limón-Mortés et al., 2008). These substrates are recognized by F-box proteins that bind consensus sequences such as D-pS/pT-G-X-X-pS and/or **D**/E/S-**S**/E/D-**G**/A-**x**_2−4_-**S**/E/D for β-TrCP (Limón-Mortés et al., 2008) (Hansen et al., 2004) or 0X000S/TPXXS/T/E for FBW7 (Limón-Mortés et al., 2008; Wertz et al., 2011) (X, 0 = random or hydrophobic amino acids, respectively). F-box proteins exhibit a high affinity for serine or threonine residues phosphorylated by specific kinases such as JNK, p38, and CKII (Limón-Mortés et al., 2008; Wertz et al., 2011). While the assembly of cullins, adaptors, and substrate receptors into multiple combinations is necessary for specific arrays of cellular biological responses through time and space, such combinatorial complexity is a major challenge for understanding CRLs' roles in cell signaling and diseases. Some substrates of CRL targeted ubiquitination and their roles are listed in Table 1.

**Table 1:** Non-exhaustive list of CRLs substrates.

**CRLs**	**Substrates**	**Receptors**	**Substrate roles**	**References**
CRL1	EMI1/Cyclin A	β-TrCP1	Regulates mitosis entry	Guardavaccaro et al., 2003
CRL1	CDC25A	β-TrCP1/2	Required for progression from G1 to the S phase of the cell cycle	Busino et al., 2003
CRL1	CDC25B	β-TrCP1/2	Required for entry into mitosis	Kanemori et al., 2005; Uchida et al., 2011
CRL1	WEE1	β-TrCP1/2	Cell cycle progression, G2/M transition	Watanabe et al., 2004
CRL1	Cyclin D1	β-TrCP1/2	Progression through the G1 phase of the cell cycle	Wei et al., 2008
CRL1	Claspin	β-TrCP1/2	Checkpoint mediated cell cycle arrest in response to replication stress and DNA damage	Peschiaroli et al., 2006
CRL1	PR-SET7/SET8	β-TrCP1/2	Epigenetic regulation/Histone modification	Wang et al., 2015
CRL1	Securin	β-TrCP	Prevent sister chromatin separation	Limón-Mortés et al., 2008
CRL1	SAK/PLK4	β-TrCP1	Prevents centrosome amplification	Cunha-Ferreira et al., 2009
CRL1	MCL1	FBXW7	Involved in apoptosis regulation	Wertz et al., 2011
CRL1	P27^KIP1^	FBXL1/SKP2	Involved in cell cycle progression	Nakayama et al., 2001
CRL1	P21^Cip1^	FBXL1/SKP2	Cell cycle progression	Bornstein et al., 2003
CRL1	P57^Kip2^	FBXL1/SKP2	Inhibitor of several G1 cyclins	Pateras et al., 2006
CRL1	P130	FBXL1/SKP2	Heterochromatin formation	Bhattacharya et al., 2003
CRL1	CDT1	FBXL1/SKP2	DNA replication licensing factor	Li et al., 2003
CRL1	Cyclin D	FBX4/FBXL1/SKP2	G1/S transition	Yu et al., 1998; Gong et al., 2014
CRL1	Cyclin G2	FBXL1/SKP2	Regulation of cell cycle progression	Xu et al., 2008
CRL1	Cyclin D2	FBXL2	Progression through the G1 phase of the cell cycle	Chen et al., 2012b
CRL1	Cyclin D3	FBXL2	G1/S transition	Chen et al., 2011
CRL1	Cyclin E	FBXW7	G1/S transition	Gong et al., 2014
CRL1	P85beta	FBXL2	Control PI3K signaling cascade	Kuchay et al., 2013
CRL1	VPS34	FBXL20	Catalytic subunit of the PI3K complex kinase	Xiao et al., 2015
CRL1	JMJD2A	FBXL4	Epigenetic regulation/Histone modification	Das et al., 2014
CRL1	CITED2	FBXL5	Transcription regulation	Machado-Oliveira et al., 2015
CRL1	Aurora A	FBXL7	Regulates mitosis	Coon et al., 2012
CRL1	Aurora B	FBXL2	Regulates mitosis	Chen B. B. et al., 2013
CRL1	CaMK1	FBXL12	Calcium/calmodulin-dependent protein kinase	Mallampalli et al., 2013
CRL1	CDC6	FBXO1/Cyclin F	DNA replication licensing factor	Walter et al., 2016
CRL1	DCAF2/CDT2	FBXO11	Efficient progression through S and G2/M phases	Abbas et al., 2013
CRL1	UHRF1	β-TrCP/FBW1A	Maintenance of DNA methylation patterns during DNA replication	Chen H. et al., 2013
CRL2	HIF1alpha	VHL	Response to hypoxia	Ohh et al., 2000
CRL2	SPRY2	VHL	May function as an antagonist to several growth factors	Anderson et al., 2011
CRL2	RNA polII subunit	VHL	Transcription	Kuznetsova et al., 2003
CRL2	CKI1	LRR1	Casein kinase involved in several cellular functions	Merlet et al., 2010
CRL2	P21^Cip1^	LRR1	Cell cycle progression	Starostina et al., 2010
CRL2	TRA1	FEM1	Epigenetic regulation/Histone modification	Shi et al., 2011
CRL2	TOPBP1	–	DNA replication	Blackford et al., 2010
CRL2	H2B	–	Core component of the nucleosome	Li et al., 2010
CRL3	NRF2	KEAP1	Negative regulation of antioxidant response	McMahon et al., 2003
CRL3	WNK4	KEAP1	Blood pressure regulation	Andérica-Romero et al., 2014
CRL3	DAXX	SPOP	Transcription repressor	Sakaue et al., 2017
CRL3	MCM3	KEAP1	DNA replication	Mulvaney et al., 2016
CRL3	PP2A	–	Resistance of cancer cells to death receptor-induced apoptosis	Xu et al., 2014
CRL3/CRL4	TOP1	–	DNA replication, transcription	Zhang et al., 2004; Kerzendorfer et al., 2010
CRL4	CDT1	DCAF2/CDT2	DNA replication licensing factor	Zhong et al., 2003; Higa et al., 2006
CRL4	P21^Cip1^	DCAF2/CDT2	Cell cycle progression	Abbas et al., 2008; Nishitani et al., 2008
CRL4	PR-SET7/SET8	DCAF2/CDT2	Epigenetic regulation	Jørgensen et al., 2011
CRL4	P27^Xic1^	DCAF2/CDT2	Cell cycle arrest	Chuang and Yew, 2001
CRL4	CKI1	DCAF2/CDT2	Casein kinase involved in several cellular functions	Kim et al., 2008
CRL4	E2F	DCAF2/CDT2	Cell cycle regulation	Shibutani et al., 2008
CRL4	TDG	DCAF2/CDT2	DNA glycosylase	Slenn et al., 2014
CRL4	CHK1	DCAF2/CDT2	Checkpoint mediated cell cycle arrest in response to DNA damage	Huh and Piwnica-Worms, 2013
CRL4	Histone H2A, H3, H4, DDB2	DDB2	Core components of the nucleosome	Kapetanaki et al., 2006
CRL4	SLBP	DCAF11	Histone biosynthesis regulation	Djakbarova et al., 2016
CRL4	CK1alpha	CRBN	Casein kinase involved in several cellular functions	Krönke et al., 2015; Petzold et al., 2016
CRL4	ZFP91	CRBN	E3 ubiquitin protein ligase	An et al., 2017b
CRL4	APP	CRBN	Cell surface receptor	Del Prete et al., 2016
CRL4	IKZF1, 3	CRBN	Transcription	Krönke et al., 2014
CRL4	Merlin	DCAF1/VprBP	Probable regulator of the Salvador/Warts/Hippo (SWH) signaling pathway	Huang and Chen, 2008
CRL4	FOXM1	DCAF1/VprBP	Transcription	Wang et al., 2017
CRL4	MCM10	DCAF1/VprBP	Replication initiation factor	Kaur et al., 2012
CRL4	TSC2	FBXW5	Regulator of several GTPases	Hu et al., 2008
CRL4	MMSET	DCAF2/CDT2	Epigenetic regulation	Evans et al., 2016
CRL4	LIG I	DCAF7	DNA replication	Peng et al., 2016
CRL4	p12 subunit of DNA polymerase δ	DCAF2/CDT2	DNA replication	Zhang et al., 2013
CRL4/CRL1	CHK1	?	Checkpoint mediated cell cycle arrest in response to DNA damage	Lampert et al., 2017; Tu et al., 2017
CRL4	SLBP	WDR23/DCAF11	Stem-loop binding protein	Lampert et al., 2017
CRL4	FBH1	DCAF2/CDT2	Helicase with a role in response to stalled/damaged replication fork	Bacquin et al., 2013
CRL4	ORCA/LRWD1	?	G1/S transition. Recruits and stabilizes replication origin complexes	Shen and Prasanth, 2012
CRL4	PCNA	?	DNA replication	Lo et al., 2012
CRL4	p53	DCAF2/CDT2	Transcription/apoptosis	Banks et al., 2006
CRL5	iNOS	SOCS	Nitric oxide production	Kuang et al., 2010; Nishiya et al., 2011
CRL5	TRII	SOCS	Enhanced migration and invasion of tumor cells by SOCS silencing	Liu et al., 2015
CRL5	GHR	SOCS	Regulation of growth hormone signaling	Bullock et al., 2006
CRL5	TRAF6	SOCS	Regulation of lipopolysaccharide signaling	Zhu et al., 2016
CRL7	Cyclin D1	FBXW8	Cell cycle arrest	Okabe et al., 2006
CRL7	IRS1	FBXW8	Regulation of insulin signaling	Xu et al., 2008
CRL7	GRASP65	FBXW8	Maintenance of the Golgi apparatus integrity	Litterman et al., 2011
CRL7	EAG1	FBXW8	Potassium channel modulation	Hsu et al., 2017
CRL9	Cytochrome C	?	Promotes cell survival	Gama et al., 2014
CRL9	Survivin	?	Genome integrity maintenance	Li et al., 2014

CRLs associate with the small protein NEDD8 (Neural precursor cell expressed developmentally down-regulated protein 8) and this interaction is essential for their ubiquitin ligase activities. NEDDylation is accomplished by the sequential action of an NAE (Nedd8-activating enzyme) and a Nedd8-conjugating enzyme, UBC12 (Haas, 2007). CRLs can be deNEDDylated by the zinc-dependent metalloenzyme CSN5, a component of the COP9 signalosome (CSN) complex, which cleaves the isopeptidic bond between cullin and NEDD8 (Cope and Deshaies, 2003). NEDDylation is also regulated by CAND1 (Cullin-associated Nedd8-dissociated protein 1), which binds to unneddylated cullins, inhibiting NEDD8 conjugation and consequently resulting in inhibition of both cullin NEDDylation and CRLs activities (Duda et al., 2011).

## Functions of CRLs in DNA replication and cell cycle progression

CRLs serve key functions in the regulation of chromosome duplication, modulating crucial steps in the assembly and disassembly of the DNA replication machinery during normal growth and in response to perturbed replication. Roles for cullin-based ring E3 ligases in DNA replication and cell cycle progress ion have been recently extensively discussed elsewhere (Abbas and Dutta, 2017), and the involvement of CRLs in the early stages of DNA replication in various organisms are briefly summarized below.

The first step in the DNA replication process in all eukaryotes is the loading of the origin recognition complex (ORC) and recruitment of the MCM2-7 helicase complex by the licensing factors CDC6 and CDT1. This complex assembly occurs during late mitosis and the early G1 phase, to form an inactive pre-replication complex (pre-RC). Pre-RCs are subsequently activated by the recruitment of additional factors and by cyclin-dependent kinases (CDKs) and DBF4-dependent kinases (DDKs) (Parker et al., 2017). CRL-controlled levels and/or activities of proteins involved in pre-RCs assembly and activation are crucial for the orderly initiation of DNA replication and the prevention of re-replication.

In yeast, RTT101, the human CUL4 homolog, modulates MRC1 (human claspin homolog) interaction with the CMG (Cdc45-MCM-GINS) helicase (Buser et al., 2016). RTT101 deletion leads to reduced association of both the replicative helicase MCM and FACT, a complex that assemble or partially disassemble nucleosomes, to replication origins (Han et al., 2010). Cells lacking RTT101 are defective in DNA replication through DNA damaged sites (Zaidi et al., 2008). The yeast CDC6, crucial for pre-RC licensing is degraded in a CRL-dependent pathway (Drury et al., 1997). CRL-induced CDC6 degradation is required to prevent DNA rereplication (Ikui et al., 2012). The yeast CMG is ubiquitinated and disassembled by DIA2 (a F-box protein related to the human CUL1/F-box complex) that binds replication origins (Koepp et al., 2006; Maculins et al., 2015).

In vertebrates, CRLs demonstrate similar functions. In *Xenopus*, CUL2 is a key player during the termination of DNA replication, disassembling the CMG helicase complex (Sonneville et al., 2017). In mammalian cells, MCM3, an essential subunit of the replicative DNA helicase, is a CRL3 substrate (Mulvaney et al., 2016). Both CRL1 and CRL4 can play important roles in the regulation of pre-RC assembly by modulating the chromatin association of two essential licensing factors, CDC6 and CDT1. CDC6 is targeted for degradation by the CRL4-CDT2 and the CRL1-CyclinF complex in S phase (Clijsters and Wolthuis, 2014) and G2-M (Walter et al., 2016) respectively. Targeted degradation of CDT1 in S-phase is shared between CRL1/SKP2 (at the G1/S transition) and CRL4/CDT2 (during S-phase) (Kim and Kipreos, 2007; Pozo and Cook, 2016; Abbas and Dutta, 2017). Thus, deregulation of CRLs in the nucleus leads to CDT1 accumulation and, in turn, to DNA re-replication and genomic instability (Kim and Kipreos, 2007; Pozo and Cook, 2016). CRL4-CDT2 also mediates the degradation of the histone H4 methyltransferase SET8, an enzyme catalyzing the monomethylation on lysine 20 of histone H4 that allows the loading of the pre-RC component ORC1 and the ORC-associated protein ORCA (Beck et al., 2012). SET8 degradation is essential to prevent DNA re-replication (Abbas et al., 2010). Another protein involved in DNA replication initiation include MMSET, a histone methyltransferase degraded during S phase in a CRL4 dependent manner and necessary for the optimum association of pre-replication factors (Evans et al., 2016). The CRL4-interacting DCAF, RepID (DCAF14/PHIP) binds a subset of replication origins and is essential for initiation from those origins (Zhang Y. et al., 2016). The mechanism by which a DCAF can facilitate initiation is unclear, however recent evidence suggests that the CRL4/CUL4B complex facilitates replication licensing through a CUL4B-CDK2-CDC6 cascade, leading to the upregulation of CDK2 and protecting CDC6 from degradation (Zou Y. et al., 2013).

CRLs also control DNA replication via indirect mechanisms. Increased CDK1/2 activities, necessary for origin firing, occur in late G1 and at the G1/S transition through the CRLs-controlled degradation of CDK inhibitors such as p27, p21, and p57 (reviewed in Abbas and Dutta, 2017). Following DNA replication initiation, cell cycle progression is also controlled, in part, by the CRL1-timely degradations of the CDK positive regulators cyclin E (for S phase progression), cyclin A, cyclin D1, and WEE1 (for G2 progression) (Watanabe et al., 2004; Abbas and Dutta, 2017). Similarly, progression over mitosis is ensured through EMI degradation by the CRL1- β-TrCP1 complex, leading to increased activity of the anaphase promoting complex/cyclosome (APC/C), an E3 ubiquitin ligase utilizing the cullin-like scaffold protein APC2. Other CRL-targeted proteins associated with cell cycle progression include Claspin, PCNA, MCM10, the DNA polymerase alpha, and histones H2A, H2B, H3, H4 among others (Table 1).

## Cullin-based ring E3 ligases and cancer

Since CRLs play critical roles in a myriad of biological processes, it is reasonable to think that the deregulation of cullins and/or other CRLs components can play a major role in cancer progression. While cullin deregulation (mostly upregulation) have been observed in cancer, downregulation or suppression of some of the CRL components can lead to tumor suppression. Deregulated expression of cullins and cullin-associated factors may occur through CpGs methylation, gene coding region mutations, or promoter deletion and/or micro-RNA-induced silencing among others. Below are several examples linking deregulation of CRLs to cancer.

### CUL1-based ubiquitin ligase complexes

**Figure 2:**
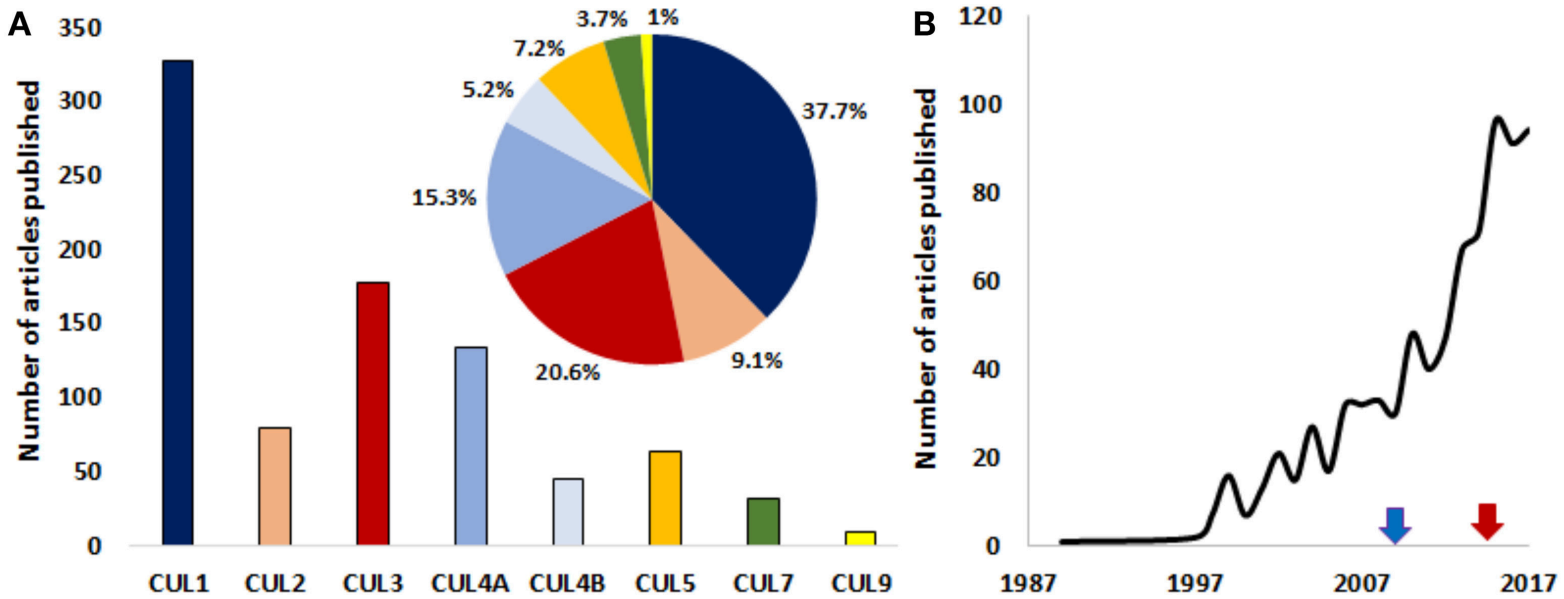
A growing interest for the roles of cullins in cancer. **(A)** Graph depicting the number of published articles studying the role of cullin1, 2, 3, 4A, 4B, 5, 7, and 9 in cancer. The pie chart shows the relative distribution (%) of published articles among the different cullins (the color code is the same as in the bar graph). **(B)** The number of published studies looking at the role of cullins and/or cullin-interacting proteins in the pathology of cancer are increasing exponentially. Blue arrow and red arrows denote the first studies targeting cullins in mice and human respectively. The number of publications for the year 2017 is an estimation made from the number of articles that were published in the first 10 months of the same year. (*Source: PubMed*).

CRL1 is the most studied cullin-associated complex in the context of cancer (Figure 2). Deregulated CUL1 expression was reported in lung and gastric cancers (Le Gallo et al., 2012) and during the early stages of melanoma development (Lee et al., 2010). A myriad of CRL1-associated F-box proteins and their substrates are involved in cancer (for a review, see Kitagawa and Kitagawa, 2016).

Altered expression and mutations in several F-box proteins, including SKP2, are hallmarks of several cancers. SKP2 (FBXL11) is part of the FBXL subfamily that comprises 22 members (FBXL1 to FBXL22) containing an F-box motif and a C-terminal Leu-rich repeat (LRR) domain. SKP2, which has been characterized as an oncoprotein, is by far the most studied F-box protein of the FBXL subfamily. SKP2 associates with the SCF (SKP1-Cullin1-F-box) complex and targets p27 for degradation, with major developmental consequences in mice (Nakayama et al., 2004). The absence of SKP2 in mice results in the accumulation of p27, nuclear enlargement, cell polyploidies, and centrosome overduplication (Nakayama et al., 2000), phenotypes that disappeared in *SKP2*^−/−^/*p27*^−/−^ double-mutant mice (Nakayama et al., 2004). Chemical-induced skin tumorigenesis is inhibited in SKP2(−/−) mice (Sistrunk et al., 2013) whereas overexpression of *Skp2* in mice led to tumor development in the prostate (Shim et al., 2003), suggesting that a SKP2 deregulation-induced oncogenesis may be tissue specific. SKP2 is also a crucial mediator of BCR-ABL-induced leukemogenesis (Agarwal et al., 2008). SKP2 is deregulated and correlated with poor prognosis in a wide array of human cancers including breast, prostate, colon, lung, brain, gastric, and blood (Frescas and Pagano, 2008; Zheng et al., 2016). Therefore, the inhibition of SKP2 could be a novel strategy for the treatment of some human cancers.

Other FBXL proteins, also mediate the degradation of substrates involved in cell-cycle progression (Table 1). Ectopic expression of FBXL2 in transformed lung epithelia facilitates polyubiquitination and degradation of cyclin D3, leading to G2/M-phase arrest, increased frequency of apoptotic cells, and chromosomal anomalies (Chen et al., 2011). FBXL2 recognizes a canonical calmodulin-binding motif within cyclin D3 and compete with calmodulin for cyclin D3 binding (Chen et al., 2012a). It is thought that FBXL2 targets cyclin D2 for degradation to inhibit cancer cell proliferation. Several patient samples show suppressed expression of FBXL2 together with robust cyclin D2 levels in acute myelogenous leukemia and acute lymphoblastic leukemia (Chen et al., 2012b). Ectopically expressed FBXL2 significantly inhibited the growth and migration of tumorigenic cells and tumor formation in athymic nude mice (Chen et al., 2012a). FBXL2 was also shown to ubiquitinate Aurora B, an integral regulator of cytokinesis that inhibits tumorigenesis (Chen B. B. et al., 2013). FBXL3 was described as a regulator of the circadian rhythm by targeting Cryptochrome (Cry1/Cry2) proteins (Siepka et al., 2007). Since, growing evidence are pointing out that deregulation of the circadian clock plays an important role in carcinogenesis (Savvidis and Koutsilieris, 2012), one putative role for FBXL in cancer could be through the disruptions of normal circadian rhythms.

FBXL proteins are also involved in the epithelial to mesenchymal transition, which often accompanies tumor progression. For example, FBXL5 and FBXL14 inhibit cell invasiveness by targeting SNAIL1 in gastric cancer cells (Vinas-Castells et al., 2010; Wu et al., 2015; Cen et al., 2017). FBXL10 (also known as KDM2B), which contains a JmiC domain (Tsukada et al., 2006) and primarily regulates metabolic and developmental genes (Zheng et al., 2016), is involved in H2AK119 ubiquitination and histone H3K36 demethylation (Wu et al., 2013) and affects TRAIL-induced apoptosis (Ge et al., 2011). The latter observation implies that targeting FBXL10 could overcome resistance to TRAIL treatment in human cancer. Like other CRL1 interacting proteins, FBXL10 role in cancer seems to be tissue specific in humans with higher levels of FBXL10 observed in several types of cancers while it is downregulated in brain tumors (Frescas et al., 2007; Tzatsos et al., 2013). A tissue-specific relationship between FBXL10 and cancer is also observed in transgenic mice. Mice with hematopoietic stem cells overexpressing FBXL10 were shown to develop myeloid or B-lymphoid leukemia (Ueda et al., 2015) whereas FBXL10 depletion was shown to abrogate tumorigenicity in the pancreas (Tzatsos et al., 2013).

Deregulation of the F-box and WD containing protein β-TrCP is associated with several cancers, including breast, colon, pancreatic, liver, gastric, and prostate (Frescas and Pagano, 2008), and both overexpression and mutations in β-TrCPs have been reported in gastric, prostate, breast, pancreas, colon liver, and skin cancer (reviewed in Zheng et al., 2016). In line with these observations, a study using transgenic mice expressing human β-TrCP 1 targeted to epithelial cells under the control of the mouse mammary tumor virus (MMTV) showed that 38% of these mice developed mammary, ovarian, and uterine carcinomas (Kudo et al., 2004). Beyond altering cell cycle progression, β-TrCP1 and β-TrCP2 are involved in the degradation of the transcription factors SNAIL and TWIST and the extracellular matrix fibronectin, all involved in metastasis (Ray et al., 2006; Kitagawa and Kitagawa, 2016). In addition, invasion of human melanoma cells is suppressed by silymarin, a plant flavonoid, in part through β-TrCP-mediated degradation of β-catenin (Vaid et al., 2011). Further evidence linking β-TrCPs to skin cancer is the observation that degradation of IκBα and PDCD4 by β-TrCPs can contribute to the development of skin squamous carcinoma (Dorrello et al., 2006; Gu et al., 2007) while expression of a dominant negative β-TrCP in mouse epidermis confers skin proliferation and apoptosis resistance in response to UVB irradiation (Bhatia et al., 2011). The role for β-TrCPs in carcinogenesis is complex since these proteins also promotes anti-cancer activities by controlling the degradation of several pro-apoptotic proteins such as MCL-1 (Ding et al., 2007), BimEL (Dehan et al., 2009), PDCD4 (Dorrello et al., 2006), pro-caspase 3 (Tan et al., 2006).

Other F-box proteins, including FBXW7, FBXW8, and FBXW9 play roles in carcinogenesis, mainly through regulating the levels of factors involved in cell cycle progression (Table 1). FBXW7, a major tumor suppressor, negatively regulates more than a dozen of oncogenic proteins with pivotal roles in cell cycle progression, proliferation, and cell division. FBXW7 also regulates protein degradation involved in DNA damage repair, cell apoptosis and metastasis (for review, see Cheng and Li, 2012) (Table 1). The *FBXW7* 4q31.3 locus is deleted in ~30% of cancers (Knuutila et al., 1999) with a *FBXW7* mutation rate of ~6% in primary tumors (Akhoondi et al., 2007). FBXW7 mutations and deletions have been described in various type of tumor types including T-cell leukemia, stomach, pancreas, breast, colon, bladder, prostate cancer, gastric, and cholangiocarcinoma with T-cell leukemia and cholangiocarcima harboring the highest mutations rates of 31 and 35% respectively (reviewed in Cheng and Li, 2012; Zheng et al., 2016). However, mutation in *FBXW7* alone may not be sufficient for carcinogenesis since a recent study showed that both *FBXW7*and *NOTCH1* deregulation may be needed for the induction of human T-ALL (Takeishi and Nakayama, 2014). The *FBXW12* gene coding regions or promoter were found to be deleted in several epithelial ovarian cancers (De la Chesnaye et al., 2015). FBXW8 modulates cancer cell proliferation through cell-type specific cyclin D1 degradation (for review, see Zheng et al., 2016). Thus, FBXW8 is involved in the proliferation of human choriocarcinoma cells via G2/M phase transition with the regulation of CDK1, CDK2, cyclin A, cyclin B1, and p27 expression (Lin et al., 2011). FBXW8 also promotes the degradation of the hematopoietic progenitor kinase 1 (HPK1), a member of mammalian STE20-like serine/threonine kinases that is lost in >95% pancreatic cancer via proteasome-mediated degradation (Wang et al., 2014). The mouse FBXW12 homolog (FBXW15) interacts with histone acetyltransferase binding to the origin recognition complex (HBO1) to mediate its CUL1-regulated ubiquitination (Zou C. B. et al., 2013). Because HBO1 plays a crucial role in DNA replication licensing and cell proliferation, FBXW15 could control DNA replication licensing and cell proliferation.

### CUL2-based ubiquitin ligase complexes

CUL2 is the scaffold protein of the CRL2 complex, recruiting the substrate receptor von Hippel-Lindau protein (pVHL) through the dimer complex EloB and EloC (Pause et al., 1997). pVHL can also be associated to CUL5 (Okumura et al., 2016). A germline mutation in VHL is the basis of familial inheritance of von Hippel-Lindau syndrome, which is characterized by the development of cysts and tumors in multiple organ systems (reviewed in Johnson et al., 2007). Mutations in pVHL or loss of heterogeneity result in high levels of HIF proteins and VHL tumorigenesis (Cassol and Mete, 2015). Deregulation of these two protein is involved in the development of VHL-associated clear-cell renal cell carcinoma (Maynard and Ohh, 2004) with pVHL ectopic expression in VHL^−/−^ renal cell carcinoma leading to suppression of tumor formation in mice (Maynard and Ohh, 2004). HIF-1α is also often overexpressed in several other cancers (Zhong et al., 1999).

In addition to the extensively studied HIF-1α, many other CRL2 substrates have been identified (Table 1). HIF-1α triggers a transcriptional response to hypoxia, a key process critical to promote tumor progression and an important determinant of resistance to therapy (Vaupel and Mayer, 2007). Disruption of CRLs components (mutation, gene loss) associates with enrichments of HIF-target genes in several tumor types (Rowbotham et al., 2014). Still, deregulation of CRLs may not solely account for deregulated HIF-1α in cancer since HIF-1α levels are controlled by different signaling mechanisms, including regulation by the HSP90 pathway, the HIF-1 pathway and the MDM2-p53 mediated ubiquitination pathway (Rowbotham et al., 2014; Cassol and Mete, 2015; Masoud and Li, 2015). Since no CUL2 mutation was found to play a critical role in HIF-1α activation in several cancers (Park et al., 2009), it is likely that HIF-1α loss of homeostasis in cancer is mediated primarily by deregulation of cullin expression rather than cullin point mutations (Zhong et al., 1999). The silencing of another CUL2 substrate, RhoB, is a crucial step driving carcinogenesis (Huang and Prendergast, 2006). In liver cancer, RhoB is targeted for degradation via the CUL2-RBX1 complex, an important effector that drives liver carcinogenesis (Xu et al., 2015). Similarly, CUL2 silencing in HPV16 positive cervical cancer cells resulted in slow growth of xenograft tumors retarding G1-S transition of the cell cycle and favoring apoptosis (Xu et al., 2016). As observed with other cullins, CUL2 deregulation may be driven by microRNAs. For example, CUL2 overexpression in gastric cancer tissues may be driven, in part, by aberrant levels of miR-574-3p (Su et al., 2012), suggesting a role for the CUL2/miR-424 pathway in promoting growth in cancer cells.

### CUL3-based ubiquitin ligase complexes

Alterations of signaling pathways caused by the deregulation of CUL3 and/or CUL3-associated factors can give rise to cancer. For example, CUL3, in complex with the substrate adaptor Ketch-like family member 20 (KLHL20), is thought to promote cancer progression through increased ubiquitination and degradation of the Promyelocytic leukemia (PML) protein (Yuan et al., 2011). Hypoxia may exacerbate PML-KLHL20-driven carcinogenesis, since the promoter of *KLHL20* contains several hypoxia-response elements (Yuan et al., 2011) and PML is a negative regulator of HIF-1 (Bernardi et al., 2006). Thus, degradation of PML by KLHL20 would potentiate a strong induction of several hypoxia pathways. Indeed, KLHL20 expression is elevated in prostate cancer and correlates with HIF-1α upregulation, and PML downregulation (Yuan et al., 2011). It should be noted however that a study with HeLa cells suggests that HIF-2α, not HIF-1α, interacts with KLHL20 and that knockdown of KLHL20 decreased HIF-2α but not HIF-1α protein levels (Higashimura et al., 2011). Although this study did not show directly that KLHL20-mediated protection of HIF-2α from degradation involves CRL3, it suggested that both HIF-1α and HIF-2α may be controlled by CRLs in cancer.

Another Kelch-like family member, KLHL39, is down regulated in cancer and correlates with both low PML expression and cancer progression (Chen et al., 2015). Unlike KLHL20, KLHL39 does not bind CUL3 but acts as an inhibitor by blocking KLHL20-mediated ubiquitination of PML by inhibiting KLHL20 binding to both CUL3 and its putative substrates (Chen et al., 2015). Thus, KLHL39 may act as a tumor suppressor by blocking KLHL20-dependent ubiquitination of PML and other substrates (Yuan et al., 2011).

The CRL3 substrate Kelch-like ECH-associated protein (KEAP1) is a key inhibitor of the transcription factor NRF2 that regulates genes involved in the antioxidant response and drugs detoxification (Chen and Chen, 2016). The CUL3-KEAP1-NRF2 pathway prevents oxidative stress-induced DNA damage and carcinogenesis in normal cells and mediates the response to oxidative stress, cell growth, and survival in cancer cells. The CRL3-KEAP1-NRF2 pathway contributions to cancer development are reinforced by the observed deregulation of KEAP1 and the presence of CUL3 mutations that could lead to NRF2 overexpression in many cancers (Chen and Chen, 2016 and references therein).

Other dual adaptor/receptor BTB proteins may have a crucial role in maintaining specific metabolic pathways controlled by hormone receptors (Zhuang et al., 2009). Speckle type BTB/POZ protein (SPOP) is one of the highest loci to exhibit loss of heterozygosity in breast cancers (Li et al., 2011) and has high mutation rates in prostate (Kan et al., 2010) and endometrial cancers (Le Gallo et al., 2012). Frequent mutations in SPOP occur in domains that interfere with E3 substrate binding and may affect SPOP's ability to degrade androgen receptors that contribute to cancer development in prostate cancer (An et al., 2014) and progesterone receptors in breast cancer cells (Gao et al., 2015).

### CUL4-based ubiquitin ligase complexes

Deregulation of CUL4A leads to tumorigenesis in transgenic mice (Jia et al., 2017) and the CUL4A locus is often amplified in many human cancers, including hepatocellular carcinomas, pleural mesotheliomas, breast and prostate cancers, squamous cell carcinoma, adrenocortical carcinoma, medulloblastoma, and ovarian invasive carcinoma (Sharma and Nag, 2014 and references therein). CUL4A overexpression in cancer is associated with tumor size, cell proliferation, migration, invasion, and cancer aggressiveness (Song et al., 2015; Deng et al., 2016; Ren et al., 2016; Jia et al., 2017; Nagel et al., 2017). In addition, CUL4A silencing can inhibit cell proliferation and invasion, and induce cell apoptosis. These processes are concomitant with increased expression of p53 and p27 and decreased expression of the metastasis—associated matrix metalloproteinase MMP-2 (Song et al., 2015). CUL4A involvement in tumorigenesis may be directly linked to its pivotal roles in the degradation of tumor suppressors or proto-oncogenic proteins associated with growth regulation, including p21, p73, p150/Sal2 and RASSF1A, N-and c-Myc and c-Jun (Sharma and Nag, 2014; Song et al., 2015). CUL4A may also play a crucial role in the regulation of PAQR3 (progestin and adipoQ receptor family member III), a newly discovered tumor suppressor that exerts its biological function through negative regulation of the oncogenic Raf/MEK/ERK signaling (Qiao et al., 2015).

Overexpression of CUL4B in several cancers such as lung, colon, pancreatic, esophageal, liver, kidney, bladder, and cervical cancer, generally associated with poor patient prognosis, has been reviewed elsewhere recently (Li and Wang, 2017). The critical role for CUL4B in tumorigenesis can be explained by its pleiotropic roles in cellular mechanisms such as cell cycle progression, DNA damage repair and apoptosis (see Li and Wang, 2017 for more details). In cervical carcinoma, CUL4B expression has been shown to be linked to histological grades with high expression related to tumor size, invasion, and metastasis (Yang et al., 2015; Jia et al., 2017; Li and Wang, 2017).

### CUL5-based ubiquitin ligase complexes

Like the CUL3-BTB complex, CUL5 may be involved in hormone receptor homeostasis. CUL5 overexpression led to decreased cell proliferation of T47D breast cancer cells (Burnatowska-Hledin et al., 2004) and attenuates estrogen receptor alpha and estrogen-dependent growth in a MAPK-dependent manner (Johnson et al., 2007). Similarly, CUL5 is significantly decreased in endometrial cancers with the most aggressive type of cancer displaying the highest CUL5 reduction (Devor et al., 2016). In concordance, CUL5 overexpression led to significantly slower growth in some endometrial cancer cells. CUL5 expression is negatively regulated by miR-19a and miR-19b (Xu et al., 2012), which are highly expressed in cervical cancer cells and are important determinants of the malignant phenotype in those cells, a phenotype that was suppressed when CUL5 3′UTR was deleted. CUL5 is also a direct target of miR-7 in liver cancer through direct miR-7 binding to the CUL5 3′UTR (Ma et al., 2013). CUL5 silencing by miR-7 led to cell cycle arrest and suppression of colony formation, suggesting that the role of CUL5 downregulation in carcinogenesis could be tissue specific.

### CUL7-based ubiquitin ligase complexes

CUL7 was first identified as a novel antiapoptotic oncogene associated with the regulation of p53 levels (Kim et al., 2007). Breast cancers can overexpress CUL7, leading to p53 downregulation (Guo et al., 2014; Men et al., 2014). CUL7 was also identified as a gene involved in liver carcinogenesis through cirrhosis associated with non-alcoholic fatty liver disease, a disease connected with metabolic syndrome. CUL7 maps to the 6p21.1 amplicon characteristic of this type of liver cancer, suggesting that this particular cancer is driven by the anti-apoptotic effect of increased CUL7 through p53 downregulation (Paradis et al., 2013). CUL7 also promotes epithelial-mesenchymal transformation of liver cancer and its high expression in liver tumors is associated with poor prognosis (Zhang D. H. et al., 2016; An et al., 2017a).

### CUL9-based ubiquitin ligase complexes

CUL9 (formerly known as PARC) is a cytoplasmic, p53-binding protein, and a p53-dependent tumor suppressor in mice (Pei et al., 2011) as well as in murine and human leukemic cells (Seipel et al., 2016; Li and Xiong, 2017). CUL9 deletion-induced tumorigenesis tends to be organ specific since mice lacking CUL9 were shown to develop tumors in sarcoma, lung, liver, and ovary only. CUL9's role in protecting genome integrity and tumor suppression is facilitated by mediating the degradation of survivin and cytochrome C in normal and cancer cells (Gama et al., 2014; Li et al., 2014).

## Targeting CRL complexes in cancer therapies

Since cullins are overexpressed in many cancer types, many novel cancer therapy strategies aim to inhibit cullin-ring ligase activity. MLN4924 (pevonedistat), a selective inhibitor of NEDD8-activating enzyme (NAE) structurally related to adenosine 5′ monophosphate that inhibits cullin NEDDylation and CRLs activity, was first shown to inhibit the growth of human colon tumor xenografts in nude mice (Soucy et al., 2009). In promising experiments, the drug was able to induce rereplication and permanent growth arrest in melanoma cells but not in immortalized non-transformed melanocytes (Benamar et al., 2016). Another NEDDylation inhibitor, TAS4464, is also tested in clinical trials (Table 2). NEDDylation inhibitors inactivate CRL E3 ubiquitin ligases and causes the cellular buildup of many substrates involves in different cellular functions (see Oladghaffari et al., 2016 for a review). Most clinical studies involving pevonedistat/MLN4924 or TAS4464 (Table 2) are still restricted to phase I and II trials.

**Table 2:** A non-exhaustive list of clinical studies targeting cullin-RING ubiquitin E3 ligases.

**Condition**	**Drug(s)**	**Measurements**	**Phase**	**References or ClinicalTrials.gov identifier**
Advanced solid tumors, neoplasms	(14C)-Pevonedistat	cumulative excretion of radioactive Pevonedistat in urine and feces/Circulatory and excretory pevonedistat metabolites Report of TEAEs and SAEs	I	NCT03057366
Recurrent AML, therapy-induced AML, untreated or recurrent AML	Pevonedistat plus Decitabine	Safety and tolerability of Pevonedistat added to Decitabine MTD of pevonedistat in combination to Decitabine mIR-155 expression, promoter methylation, and mIR-155 target gene expression (SHIP1/PU.1) NF-kappaB expression and enrichment on mIR-155 promoter	I	NCT03009240
Metastatic melanoma	Pevonedistat	MTD of 209 mg/m^2^ Clinical activity: 3% PR, 48% *SD* Pevonedistat plasma concentration increased approximately proportionally with dose from 50 to 278 mg/m^2^ after Day 1 intravenous infusion	I	NCT01011530 (^*^) (Bhatia et al., 2016)
Solid tumors	MLN4924 plus Docetaxel 2.]MLN4924 plus Docetaxel plus Carboplatin MLN4924 plus Gemcitabine	Number of adverse events Time course MLN4924 plasma concentration	I	NCT01862328
Advanced solid tumors	MLN4924 (schedules A and C) MLN4924 + Dexamethasone (Schedule B)	MTD of 50 mg/m^2^ (schedule A) 50 and 67 mg/m^2^ (schedule B and C, respectively) 11/13 patients with > 20% increase in CDT1 and NRF2 CRLs substrates 13/14 patients show NEDD8 adducts in tumor biopsies Clinical activity: 74% SD for schedules B and C	I	NCT00677170 (^*^) (Sarantopoulos et al., 2016)
AML	MLN4924 plus Azacitidine	Safety and tolerability of MLN4924 in combination with Azacitidine Disease response rate 30-day and 60-day mortality rate	I	NCT01814826
Advanced solid tumors	MLN4924 Fluconazole Itraconazole Docetaxel Carboplatin Paclitaxel	TEAEs and disease response MLN4924 plasma concentration, blood to plasma ratio. MLN4924 clearance Clinical response	I	NCT02122770
AML, ALL, MDS	MLN4924 Intravenous infusion on days 1, 3, and 5 (schedule A) and 1, 4, 8, and 11 (schedule B)	MTD of 59 (Schedule A) and 83 mg/m^2^ (Schedule B) Clinical activity: 17% CR/PR (schedule A); 10% CR/PR (schedule B) 32/35 patients with NEDD8 adduct in tumor biopsies Pevonedistat increased within 4–8 h after infusion and returned to baseline within 24 h	I	NCT00911066 (^*^) (Swords et al., 2015)
Leukemia, MDS, Myeloid, Acute	Pevonedistat Pevonedistat plus Azacitidine	TEAEs and dose limiting toxicities Overall and complete responses Pevonedistat plasma concentration and clearance	I	NCT02782468
Relapsed/refractory multiple Myeloma or lymphoma	MLN4924 Intravenous infusion on Week 1, 2, 8, and 9 (schedule A) and 1, 4, 8, and 11 (schedule B)	MTD of 110 mg/m^2^ (schedule A) and 196 mg/m^2^ (schedule B) 11/13 patients with NEDD8 adducts in bone marrow aspirates CDT1 and NRF2 skin and NRF2 mRNA in blood increased in treated patients Clinical activity: 1 patient with PR and 71% SD	I	NCT00722488 (^*^) (Shah et al., 2016)
Multiple myeloma Non-Hodgkin lymphoma	TAS4464	Investigate the safety and tolerability of TAS4464; identify TAS4464 MTD Efficacy of TAS4464, defined as Objective Response Rate (ORR) per IWG criteria (NHL) and IMWG criteria (MM).	I II	NCT02978235
MDS leukemia, CML	Azacitidine Azacitidine plus Pevonedistat	EVF OS	II	NCT02610777
Non-small cell lung cancer	Pevonedistat plus Docetaxel	Response to treatment Median progression free survival time, OS time, and patients who achieve stable disease Toxicities by system organ class	II	NCT03228186
MDS leukemia, CML, AML	Azacytidine Azacytidine plus Pevonedistat	EVF, OS, partial remission overall response. 6 months and 1 year survival rate	II	NCT02610777
MDS leukemia, CML	Azacitidine Azacitidine plus Pevonedistat	Overall response and EVF OS Pevonedistat plasma concentration EVF and OS in participants with TP53 mutations or any adverse cytogenetic risk group	III	NCT03268954

*EVF, Event-Free Survival; OS, Overall Survival; AML, Acute Myeloid Leukemia; CML, Chronic Myelomonocytic Leukemia. TEAEs, Treatment Emergent Adverse Events; SAEs, Serious Adverse Events; MDS, Myelodysplastic Syndrome; MTD, maximum tolerated dose. CR, complete response; PR, partial response; SD, Stable diseases*.

(*) *Study completed*.

As shown in Table 2, the first phase I study involving a NEDDylation inhibitor (pevonedistat) investigated both pharmacokinetics and pharmacodynamics in patients with acute myeloid leukemia and myelodysplastic syndromes and demonstrated a modest clinical activity (Swords et al., 2015). Subsequent phase I studies evaluated the use of pevonedistat against relapsed/refractory multiple myeloma or lymphoma (Shah et al., 2016), advanced nonhematologic malignancies (Sarantopoulos et al., 2016), and metastatic melanoma (Bhatia et al., 2016). At the time of writing, there are 11 ongoing clinical trials using MLN4294/pevonedistat targeting both solid tumors (4) and blood cancers (7) (Table 1 and clinicaltrials.gov). Since MLN4924 sensitizes cancer cells to several chemotherapeutic drugs (reviewed in Oladghaffari et al., 2016), the majority of ongoing trials (10/11) are evaluating pevonedistat in combination with other anti-tumor drugs such as DNA damaging agents such as carboplatin, nucleoside analogs (azacitidine, gemcitabine, decitabine), and tubulin-binding drugs (paclitaxel, vincristine). In many studies, NAE inhibition by pevonedistat was confirmed *in vivo* by the accumulation of cullin-ring ligases substrates, including CDT1 and NRF2 in solid tumors and upregulation of NRF2 gene in blood. In the metastatic melanoma study (Bhatia et al., 2016), an additional panel of NAE-regulated substrates (ATF3, GCLM, GSR, MAG1, NQO1, SLC7A11, SRXN1, TXNRD1) was used to confirm inhibition of NAE in blood and increases in pevonedistat–NEDD8 adducts. CDT1 and NRF2 protein levels were measured in tumor biopsies. In the study related to advanced nonhematologic malignancies, stable disease was observed in 80% of the patients receiving both dexamethasone and pevonedistat, and in 69% of patients receiving pevonedistat alone (Sarantopoulos et al., 2016). In patients with metastatic melanoma (Bhatia et al., 2016), one patient (3%) achieved partial response while 15 patients (48%) showed stable disease.

Several studies have shown the proof of concept by using MLN4924 for increased cancer cell killing by radiation. MLN4924 sensitized head and neck squamous carcinoma cells to ionizing radiation and enhances radiation-induced suppression of xenografts in mice (Vanderdys et al., 2017). MLN4924 also enhanced the susceptibility of nasopharyngeal carcinoma, colorectal, lung, pancreatic, and breast cancer cells to radiation (Oladghaffari et al., 2016, 2017; Wan et al., 2016; Xie et al., 2017). Importantly, MLN4924 was shown to sensitize several types of cancer cells to ionizing radiation with a minimal effect on non-cancerous cells (Wei et al., 2012). Mechanistically, MLN4924-increased radiosensitization may be due to induced G2 cell arrest, apoptosis, delayed DNA repair, and loss of radical oxygen species homeostasis (Oladghaffari et al., 2016; Wang et al., 2016). For all these reasons, future clinical trials may expand the use of NAE inhibitors to radiotherapy to treat cancer.

## Future directions

Developing a better understanding of the contributions of each Cullin-Ring Ligase complex in cellular homeostasis remains a challenging task. A large portion of the 100,000 different proteins that are present per cell needs to be recycled or eliminated in a timely manner during development or cell cycle progression. The complexity of such a task explains the extreme intricacies of protein degradation, where substrate recognition (or protein modification recognition) is crucial. It also suggests that most of the CRL substrates are yet to be discovered. Future studies are expected to reveal new CRL-interacting factors and new regulatory pathways, and provide further insights into the existence of regulatory crosstalk among the different CRLs. New roles for cullins in carcinogenesis will assuredly emerge in the near future since the relationship between cancer and some cullins (i.e., CUL7 and CUL9) is still a relatively new concept (Figure 2).

While drugs that inhibit all CRLs are currently being validated, it is plausible that future drug development will also target individual CRLs or specific CRLs-interacting factors. For example, a specific SKP2 inhibitor that selectively suppresses the CRL1 E3 ligase activity was reported to exhibit anticancer activity against human tumor xenografts in mice (Chan et al., 2013). In another approach, homo-bivalent molecules aiming to target CRL2-VHL induced preferential dimerization and isoform-selective degradation of VHL (Maniaci et al., 2017). Further development of agents that modulate specific interactions with substrate receptors is expected in the future. Of particular interest are CRLs that play regulatory roles in molecular pathways altered in cancer cells, such as components of chromatin-associated CRLs that modulate DNA replication. Such a development will benefit from further understanding of the interactions that fine-tune DNA replication, such as selective interactions of groups of replication origins with distinct regulatory proteins, including DCAF members of CRL4 (Zhang Y. et al., 2016; Aladjem and Redon, 2017). Future advances will be likely target CRL-mediated pathways that maintain genomic stability by preventing DNA rereplication and modulate the S-phase DNA damage response via protein degradation.

## Author contributions

All authors listed have made a substantial, direct and intellectual contribution to the work, and approved it for publication.

### Conflict of interest statement

The authors declare that the research was conducted in the absence of any commercial or financial relationships that could be construed as a potential conflict of interest.

## References

[B1] Abbas T. Dutta A. (2017). Regulation of mammalian DNA replication via the ubiquitin-proteasome system. Adv. Exp. Med. Biol. 1042, 421–454. 10.1007/978-981-10-6955-0_19 29357069 PMC5933532

[B2] Abbas T. Keaton M. Dutta A. (2013). Regulation of TGF-beta signaling, exit from the cell cycle, and cellular migration through cullin cross-regulation: SCF-FBXO11 turns off CRL4-Cdt2. Cell Cycle 12, 2175–2182. 10.4161/cc.25314 23892434 PMC3755067

[B3] Abbas T. Shibata E. Park J. Jha S. Karnani N. Dutta A. (2010). CRL4(Cdt2) regulates cell proliferation and histone gene expression by targeting PR-Set7/Set8 for degradation. Mol. Cell 40, 9–21. 10.1016/j.molcel.2010.09.014 20932471 PMC2966975

[B4] Abbas T. Sivaprasad U. Terai K. Amador V. Pagano M. Dutta A. (2008). PCNA-dependent regulation of p21 ubiquitylation and degradation via the CRL4Cdt2 ubiquitin ligase complex. Genes Dev. 22, 2496–2506. 10.1101/gad.1676108 18794347 PMC2546691

[B5] Agarwal A. Bumm T. G. Corbin A. S. O'Hare T. Loriaux M. VanDyke J.. (2008). Absence of SKP2 expression attenuates BCR-ABL-induced myeloproliferative disease. Blood 112, 1960–1970. 10.1182/blood-2007-09-113860 18559973 PMC2518897

[B6] Akhoondi S. Sun D. von der Lehr N. Apostolidou S. Klotz K. Maljukova A.. (2007). FBXW7/hCDC4 is a general tumor suppressor in human cancer. Cancer Res. 67, 9006–9012. 10.1158/0008-5472.CAN-07-1320 17909001

[B7] Aladjem M. I. Redon C. E. (2017). Order from clutter: selective interactions at mammalian replication origins. Nat. Rev. Genet. 18, 101–116. 10.1038/nrg.2016.141 27867195 PMC6596300

[B8] An J. Liu Z. Liang Q. Pan Y. Li H. Wang R.. (2017a). Overexpression of RabL3 and Cullin7 is associated with pathogenesis and poor prognosis in hepatocellular carcinoma. Hum. Pathol. 67, 146–151. 10.1016/j.humpath.2017.07.008 28739496

[B9] An J. Ponthier C. M. Sack R. Seebacher J. Stadler M. B. Donovan K. A.. (2017b). pSILAC mass spectrometry reveals ZFP91 as IMiD-dependent substrate of the CRL4CRBN ubiquitin ligase. Nat. Commun. 8:15398. 10.1038/ncomms15398 28530236 PMC5458144

[B10] An J. Wang C. Deng Y. Yu L. Huang H. (2014). Destruction of full-length androgen receptor by wild-type SPOP, but not prostate-cancer-associated mutants. Cell Rep. 6, 657–669. 10.1016/j.celrep.2014.01.013 24508459 PMC4361392

[B11] Andérica-Romero A. C. Escobar L. Padilla-Flores T. Pedraza-Chaverri J. (2014). Insights in cullin 3/WNK4 and its relationship to blood pressure regulation and electrolyte homeostasis. Cell. Signal. 26, 1166–1172. 10.1016/j.cellsig.2014.01.032 24518042

[B12] Anderson K. Nordquist K. A. Gao X. Hicks K. C. Zhai B. Gygi S. P.. (2011). Regulation of cellular levels of Sprouty2 protein by prolyl hydroxylase domain and von Hippel-Lindau proteins. J. Biol. Chem. 286, 42027–42036. 10.1074/jbc.M111.303222 22006925 PMC3234935

[B13] Bacquin A. Pouvelle C. Siaud N. Perderiset M. Salomé-Desnoulez S. Tellier-Lebegue C.. (2013). The helicase FBH1 is tightly regulated by PCNA via CRL4(Cdt2)-mediated proteolysis in human cells. Nucleic Acids Res. 41, 6501–6513. 10.1093/nar/gkt397 23677613 PMC3711418

[B14] Banks D. Wu M. Higa L. A. Gavrilova N. Quan J. Ye T.. (2006). L2DTL/CDT2 and PCNA interact with p53 and regulate p53 polyubiquitination and protein stability through MDM2 and CUL4A/DDB1 complexes. Cell Cycle 5, 1719–1729. 10.4161/cc.5.15.3150 16861890

[B15] Beck D. B. Burton A. Oda H. Ziegler-Birling C. Torres-Padilla M. E. Reinberg D. (2012). The role of PR-Set7 in replication licensing depends on Suv4-20h. Genes Dev. 26, 2580–2589. 10.1101/gad.195636.112 23152447 PMC3521623

[B16] Benamar M. Guessous F. Du K. Corbett P. Obeid J. Gioeli D.. (2016). Inactivation of the CRL4-CDT2-SET8/p21 ubiquitylation and degradation axis underlies the therapeutic efficacy of pevonedistat in melanoma. EBioMedicine 10, 85–100. 10.1016/j.ebiom.2016.06.023 27333051 PMC5006603

[B17] Bernardi R. Guernah I. Jin D. Grisendi S. Alimonti A. Teruya-Feldstein J. (2006). PML inhibits HIF-1 alpha translation and neoangiogenesis through repression of mTOR. Nature 442, 779–785. 10.1038/nature05029 16915281

[B18] Bhatia N. Demmer T. A. Sharma A. K. Elcheva I. Spiegelman V. S. (2011). Role of beta-TrCP ubiquitin ligase receptor in UVB mediated responses in skin. Arch. Biochem. Biophys. 508, 178–184. 10.1016/j.abb.2010.12.023 21187057 PMC3150842

[B19] Bhatia S. Pavlick A. C. Boasberg P. Thompson J. A. Mulligan G. Pickard M. D.. (2016). A phase I study of the investigational NEDD8-activating enzyme inhibitor pevonedistat (TAK-924/MLN4924) in patients with metastatic melanoma. Invest. New Drugs 34, 439–449. 10.1007/s10637-016-0348-5 27056178 PMC4919369

[B20] Bhattacharya S. Garriga J. Calbó J. Yong T. Haines D. S. Grana X. (2003). SKP2 associates with p130 and accelerates p130 ubiquitylation and degradation in human cells. Oncogene 22, 2443–2451. 10.1038/sj.onc.1206339 12717421

[B21] Blackford A. N. Patel R. N. Forrester N. A. Theil K. Groitl P. Stewart G. S.. (2010). Adenovirus 12 E4orf6 inhibits ATR activation by promoting TOPBP1 degradation. Proc. Natl. Acad. Sci. U.S.A. 107, 12251–12256. 10.1073/pnas.0914605107 20566845 PMC2901489

[B22] Bornstein G. Bloom J. Sitry-Shevah D. Nakayama K. Pagano M. Hershko A. (2003). Role of the SCFSkp2 ubiquitin ligase in the degradation of p21Cip1 in S phase. J. Biol. Chem. 278, 25752–25757. 10.1074/jbc.M301774200 12730199

[B23] Bradsher J. N. Jackson K. W. Conaway R. C. Conaway J. W. (1993). RNA polymerase II transcription factor SIII. I. Identification, purification, and properties. J. Biol. Chem. 268, 25587–25593. 8244996

[B24] Bulatov E. Ciulli A. (2015). Targeting Cullin-RING E3 ubiquitin ligases for drug discovery: structure, assembly and small-molecule modulation. Biochem. J. 467, 365–386. 10.1042/BJ20141450 25886174 PMC4403949

[B25] Bullock A. N. Debreczeni J. E. Edwards A. M. Sundström M. Knapp S. (2006). Crystal structure of the SOCS2-elongin C-elongin B complex defines a prototypical SOCS box ubiquitin ligase. Proc. Natl. Acad. Sci. U.S.A. 103, 7637–7642. 10.1073/pnas.0601638103 16675548 PMC1472497

[B26] Burnatowska-Hledin M. A. Kossoris J. B. Van Dort C. J. Shearer R. L. Zhao P. Murrey D. A.. (2004). T47D breast cancer cell growth is inhibited by expression of VACM-1, a cul-5 gene. Biochem. Biophys. Res. Commun. 319, 817–825. 10.1016/j.bbrc.2004.05.057 15184056

[B27] Buser R. Kellner V. Melnik A. Wilson-Zbinden C. Schellhaas R. Kastner L.. (2016). The replisome-coupled E3 ubiquitin ligase Rtt101Mms22 counteracts Mrc1 function to tolerate genotoxic stress. PLoS Genet. 12:e1005843. 10.1371/journal.pgen.1005843 26849847 PMC4743919

[B28] Busino L. Donzelli M. Chiesa M. Guardavaccaro D. Ganoth D. Dorrello N. V.. (2003). Degradation of Cdc25A by beta-TrCP during S phase and in response to DNA damage. Nature 426, 87–91. 10.1038/nature02082 14603323

[B29] Cardote T. A. F. Gadd M. S. Ciulli A. (2017). Crystal structure of the Cul2-Rbx1-EloBC-VHL ubiquitin ligase complex. Structure 25, 901 e903–911 e903. 10.1016/j.str.2017.04.009 28591624 PMC5462531

[B30] Cassol C. Mete O. (2015). Endocrine manifestations of von Hippel-Lindau disease. Arch. Pathol. Lab. Med. 139, 263–268. 10.5858/arpa.2013-0520-RS 25611110

[B31] Cen G. Ding H. H. Liu B. (2017). FBXL5 targets Cortactin for ubiquitination-mediated destruction to regulate gastric cancer cell migration. Tumor Biol. 35, 8633–8638. 10.1007/s13277-014-2104-9 24867096

[B32] Chan C. H. Morrow J. K. Li C. F. Gao Y. Jin G. Moten A.. (2013). Pharmacological inactivation of Skp2 SCF ubiquitin ligase restricts cancer stem cell traits and cancer progression. Cell 154, 556–568. 10.1016/j.cell.2013.06.048 23911321 PMC3845452

[B33] Chen B. B. Glasser J. R. Coon T. A. Mallampalli R. K. (2011). FBXL2 is a ubiquitin E3 ligase subunit that triggers mitotic arrest. Cell Cycle 10, 3487–3494. 10.4161/cc.10.20.17742 22024926 PMC3266178

[B34] Chen B. B. Glasser J. R. Coon T. A. Mallampalli R. K. (2012a). F-box protein FBXL2 exerts human lung tumor suppressor-like activity by ubiquitin-mediated degradation of cyclin D3 resulting in cell cycle arrest. Oncogene 31, 2566–2579. 10.1038/onc.2011.432 22020328 PMC3266958

[B35] Chen B. B. Glasser J. R. Coon T. A. Mallampalli R. K. (2013). Skp-cullin-F box E3 ligase component FBXL2 ubiquitinates Aurora B to inhibit tumorigenesis. Cell Death Dis. 4:e759. 10.1038/cddis.2013.271 23928698 PMC3763433

[B36] Chen B. B. Glasser J. R. Coon T. A. Zou C. Miller H. L. Fenton M.. (2012b). F-box protein FBXL2 targets cyclin D2 for ubiquitination and degradation to inhibit leukemic cell proliferation. Blood 119, 3132–3141. 10.1182/blood-2011-06-358911 22323446 PMC3321873

[B37] Chen H. Y. Chen R. H. (2016). Cullin 3 ubiquitin ligases in cancer biology: functions and therapeutic implications. Front. Oncol. 6:113. 10.3389/fonc.2016.00113 27200299 PMC4852199

[B38] Chen H. Y. Hu J. Y. Chen T. H. Lin Y. C. Liu X. Lin M. Y.. (2015). KLHL39 suppresses colon cancer metastasis by blocking KLHL20-mediated PML and DAPK ubiquitination. Oncogene 34, 5141–5151. 10.1038/onc.2014.435 25619834

[B39] Chen H. Ma H. Inuzuka H. Diao J. Lan F. Shi Y. G.. (2013). DNA damage regulates UHRF1 stability via the SCF(beta-TrCP) E3 ligase. Mol. Cell. Biol. 33, 1139–1148. 10.1128/MCB.01191-12 23297342 PMC3592027

[B40] Cheng Y. Li G. (2012). Role of the ubiquitin ligase Fbw7 in cancer progression. Cancer Metastasis Rev. 31, 75–87. 10.1007/s10555-011-9330-z 22124735

[B41] Chuang L. C. Yew P. R. (2001). Regulation of nuclear transport and degradation of the Xenopus cyclin-dependent kinase inhibitor, p27Xic1. J. Biol. Chem. 276, 1610–1617. 10.1074/jbc.M008896200 11044455

[B42] Clijsters L. Wolthuis R. (2014). PIP-box-mediated degradation prohibits re-accumulation of Cdc6 during S phase. J. Cell. Sci. 127(Pt 6), 1336–1345. 10.1242/jcs.145862 24434580

[B43] Coon T. A. Glasser J. R. Mallampalli R. K. Chen B. B. (2012). Novel E3 ligase component FBXL7 ubiquitinates and degrades Aurora A, causing mitotic arrest. Cell Cycle 11, 721–729. 10.4161/cc.11.4.19171 22306998 PMC3318106

[B44] Cope G. A. Deshaies R. J. (2003). COP9 signalosome: a multifunctional regulator of SCF and other cullin-based ubiquitin ligases. Cell 114, 663–671. 10.1016/S0092-8674(03)00722-0 14505567

[B45] Cunha-Ferreira I. Rodrigues-Martins A. Bento I. Riparbelli M. Zhang W. Laue E.. (2009). The SCF/Slimb ubiquitin ligase limits centrosome amplification through degradation of SAK/PLK4. Curr. Biol. 19, 43–49. 10.1016/j.cub.2008.11.037 19084407

[B46] Das A. Chai J. C. Jung K. H. Das N. D. Kang S. C. Lee Y. S.. (2014). JMJD2A attenuation affects cell cycle and tumourigenic inflammatory gene regulation in lipopolysaccharide stimulated neuroectodermal stem cells. Exp. Cell Res. 328, 361–378. 10.1016/j.yexcr.2014.08.029 25193078

[B47] De la Chesnaye E. Méndez J. P. López-Romero R. Romero-Tlalolini M. D. Vergara M. D. Salcedo M.. (2015). FBXW12, a novel F box protein-encoding gene, is deleted or methylated in some cases of epithelial ovarian cancer. Int. J. Clin. Exp. Pathol. 8, 10192–10203. 26617728 PMC4637543

[B48] Dehan E. Bassermann F. Guardavaccaro D. Vasiliver-Shamis G. Cohen M. Lowes K. N.. (2009). betaTrCP- and Rsk1/2-mediated degradation of BimEL inhibits apoptosis. Mol. Cell 33, 109–116. 10.1016/j.molcel.2008.12.020 19150432 PMC2655121

[B49] Del Prete D. Rice R. C. Rajadhyaksha A. M. D'Adamio L. (2016). Amyloid Precursor Protein (APP) May Act as a substrate and a recognition unit for CRL4CRBN and Stub1 E3 ligases facilitating ubiquitination of proteins involved in presynaptic functions and neurodegeneration. J. Biol. Chem. 291, 17209–17227. 10.1074/jbc.M116.733626 27325702 PMC5016122

[B50] Deng J. Lei W. Xiang X. Zhang L. Lei J. Gong Y.. (2016). Cullin 4A (CUL4A), a direct target of miR-9 and miR-137, promotes gastric cancer proliferation and invasion by regulating the Hippo signaling pathway. Oncotarget 7, 10037–10050. 10.18632/oncotarget.7048 26840256 PMC4891102

[B51] Devor E. J. Schickling B. M. Reyes H. D. Warrier A. Lindsay B. Goodheart M. J.. (2016). Cullin-5, a ubiquitin ligase scaffold protein, is significantly underexpressed in endometrial adenocarcinomas and is a target of miR-182. Oncol. Rep. 35, 2461–2465. 10.3892/or.2016.4605 26847831 PMC4774736

[B52] Dias D. C. Dolios G. Wang R. Pan Z. Q. (2002). CUL7: a doc domain-containing cullin selectively binds Skp1.Fbx29 to form an SCF-like complex. Proc. Natl. Acad. Sci. U.S.A. 99, 16601–16606. 10.1073/pnas.252646399 12481031 PMC139190

[B53] Ding Q. He X. Hsu J. M. Xia W. Chen C. T. Li L. Y.. (2007). Degradation of Mcl-1 by beta-TrCP mediates glycogen synthase kinase 3-induced tumor suppression and chemosensitization. Mol. Cell. Biol. 27, 4006–4017. 10.1128/MCB.00620-06 17387146 PMC1900029

[B54] Djakbarova U. Marzluff W. F. Köseoglu M. M. (2016). DDB1 and CUL4 associated factor 11 (DCAF11) mediates degradation of Stem-loop binding protein at the end of S phase. Cell Cycle 15, 1986–1996. 10.1080/15384101.2016.1191708 27254819 PMC4968976

[B55] Dorrello N. V. Peschiaroli A. Guardavaccaro D. Colburn N. H. Sherman N. E. Pagano M. (2006). S6K1- and betaTRCP-mediated degradation of PDCD4 promotes protein translation and cell growth. Science 314, 467–471. 10.1126/science.1130276 17053147

[B56] Drury L. S. Perkins G. Diffley J. F. (1997). The Cdc4/34/53 pathway targets Cdc6p for proteolysis in budding yeast. EMBO J. 16, 5966–5976. 10.1093/emboj/16.19.5966 9312054 PMC1170227

[B57] Duda D. M. Scott D. C. Calabrese M. F. Zimmerman E. S. Zheng N. Schulman B. A. (2011). Structural regulation of cullin-RING ubiquitin ligase complexes. Curr. Opin. Struct. Biol. 21, 257–264. 10.1016/j.sbi.2011.01.003 21288713 PMC3151539

[B58] Evans D. L. Zhang H. X. Ham H. Pei H. D. Lee S. Kim J.. (2016). MMSET is dynamically regulated during cell-cycle progression and promotes normal DNA replication. Cell Cycle 15, 95–105. 10.1080/15384101.2015.1121323 26771714 PMC4825781

[B59] Frescas D. Pagano M. (2008). Deregulated proteolysis by the F-box proteins SKP2 and beta-TrCP: tipping the scales of cancer. Nat. Rev. Cancer 8, 438–449. 10.1038/nrc2396 18500245 PMC2711846

[B60] Frescas D. Guardavaccaro D. Bassermann F. Koyama-Nasu R. Pagano M. (2007). JHDM1B/FBXL10 is a nucleolar protein that represses transcription of ribosomal RNA genes. Nature 450, 309–317. 10.1038/nature06255 17994099

[B61] Gama V. Swahari V. Schafer J. Kole A. J. Evans A. Huang Y.. (2014). The E3 ligase PARC mediates the degradation of cytosolic cytochrome c to promote survival in neurons and cancer cells. Sci. Signal. 7:ra67. 10.1126/scisignal.2005309 25028717 PMC4182917

[B62] Gao K. Jin X. Tang Y. Ma J. Peng J. Yu L.. (2015). Tumor suppressor SPOP mediates the proteasomal degradation of progesterone receptors (PRs) in breast cancer cells. Am. J. Cancer Res. 5, 3210–3220. 26693071 PMC4656742

[B63] Ge R. Wang Z. Zeng Q. Xu X. Olumi A. F. (2011). F-box protein 10, an NF-kappaB-dependent anti-apoptotic protein, regulates TRAIL-induced apoptosis through modulating c-Fos/c-FLIP pathway. Cell Death Differ. 18, 1184–1195. 10.1038/cdd.2010.185 21252908 PMC3131965

[B64] Gong Y. Zack T. I. Morris L. G. Lin K. Hukkelhoven E. Raheja R.. (2014). Pan-cancer genetic analysis identifies PARK2 as a master regulator of G1/S cyclins. Nat. Genet. 46, 588–594. 10.1038/ng.2981 24793136 PMC4251771

[B65] Gu Q. Y. Bowden G. T. Normolle D. Sun Y. (2007). SAG/ROC2 E3 ligase regulates skin carcinogenesis by stage-dependent targeting of c-jun/AP1 and I kappa B-alpha/NF-kappa B. J. Cell Biol. 178, 1009–1023. 10.1083/jcb.200612067 17846172 PMC2064624

[B66] Guardavaccaro D. Kudo Y. Boulaire J. Barchi M. Busino L. Donzelli M.. (2003). Control of meiotic and mitotic progression by the F box protein beta-Trcp1 *in vivo*. Dev. Cell 4, 799–812. 10.1016/S1534-5807(03)00154-0 12791266

[B67] Guo H. S. Wu F. Wang Y. Yan C. Su W. (2014). Overexpressed ubiquitin ligase Cullin7 in breast cancer promotes cell proliferation and invasion via down-regulating p53. Biochem. Biophys. Res. Commun. 450, 1370–1376. 10.1016/j.bbrc.2014.06.134 25003318

[B68] Haas A. L. (2007). Structural insights into early events in the conjugation of ubiquitin and ubiquitin-like proteins. Mol. Cell 27, 174–175. 10.1016/j.molcel.2007.07.003 17643365

[B69] Han J. Li Q. McCullough L. Kettelkamp C. Formosa T. Zhang Z. (2010). Ubiquitylation of FACT by the cullin-E3 ligase Rtt101 connects FACT to DNA replication. Genes Dev. 24, 1485–1490. 10.1101/gad.1887310 20634314 PMC2904938

[B70] Hansen D. V. Loktev A. V. Ban K. H. Jackson P. K. (2004). Plk1 regulates activation of the anaphase promoting complex by phosphorylating and triggering SCFbetaTrCP-dependent destruction of the APC Inhibitor Emi1. Mol. Biol. Cell 15, 5623–5634. 10.1091/mbc.E04-07-0598 15469984 PMC532041

[B71] Harper J. W. Tan M. K. (2012). Understanding cullin-RING E3 biology through proteomics-based substrate identification. Mol. Cell. Proteomics 11, 1541–1550. 10.1074/mcp.R112.021154 22962057 PMC3518111

[B72] Higa L. A. Banks D. Wu M. Kobayashi R. Sun H. Zhang H. (2006). L2DTL/CDT2 interacts with the CUL4/DDB1 complex and PCNA and regulates CDT1 proteolysis in response to DNA damage. Cell Cycle 5, 1675–1680. 10.4161/cc.5.15.3149 16861906

[B73] Higashimura Y. Terai T. Yamaji R. Mitani T. Ogawa M. Harada N.. (2011). Kelch-like 20 up-regulates the expression of hypoxia-inducible factor-2alpha through hypoxia- and von Hippel-Lindau tumor suppressor protein-independent regulatory mechanisms. Biochem. Biophys. Res. Commun. 413, 201–205. 10.1016/j.bbrc.2011.08.058 21888897

[B74] Hsu P. H. Ma Y. T. Fang Y. C. Huang J. J. Gan Y. L. Chang P. T.. (2017). Cullin 7 mediates proteasomal and lysosomal degradations of rat Eag1 potassium channels. Sci. Rep. 7:40825. 10.1038/srep40825 28098200 PMC5241692

[B75] Hu J. Zacharek S. He Y. J. Lee H. Shumway S. Duronio R. J.. (2008). WD40 protein FBW5 promotes ubiquitination of tumor suppressor TSC2 by DDB1-CUL4-ROC1 ligase. Genes Dev. 22, 866–871. 10.1101/gad.1624008 18381890 PMC2279197

[B76] Huang J. Chen J. (2008). VprBP targets Merlin to the Roc1-Cul4A-DDB1 E3 ligase complex for degradation. Oncogene 27, 4056–4064. 10.1038/onc.2008.44 18332868

[B77] Huang M. Prendergast G. C. (2006). RhoB in cancer suppression. Histol. Histopathol. 21, 213–218. 10.14670/HH-21.213 16329046

[B78] Huh J. Piwnica-Worms H. (2013). CRL4(CDT2) targets CHK1 for PCNA-independent destruction. Mol. Cell. Biol. 33, 213–226. 10.1128/MCB.00847-12 23109433 PMC3554108

[B79] Ikui A. E. Rossio V. Schroeder L. Yoshida S. (2012). A yeast GSK-3 kinase Mck1 promotes Cdc6 degradation to inhibit DNA re-replication. PLoS Genet. 8:e1003099. 10.1371/journal.pgen.1003099 23236290 PMC3516531

[B80] Jackson P. K. (2014). Regulating microtubules and genome stability via the CUL7/3M syndrome complex and CUL9. Mol. Cell 54, 713–715. 10.1016/j.molcel.2014.05.024 24905004

[B81] Jia L. Yan F. Cao W. Chen Z. Zheng H. Li H.. (2017). Dysregulation of CUL4A and CUL4B ubiquitin ligases in lung cancer. J. Biol. Chem. 292, 2966–2978. 10.1074/jbc.M116.765230 27974468 PMC5314191

[B82] Johnson A. E. Le I. P. Buchwalter A. Burnatowska-Hiedin M. A. (2007). Estrogen-dependent growth and estrogen receptor (ER)-alpha concentration in T47D breast cancer cells are inhibited by VACM-1, a cul 5 gene. Mol. Cell. Biochem. 301, 13–20. 10.1007/s11010-006-9392-3 17186378

[B83] Jørgensen S. Eskildsen M. Fugger K. Hansen L. Larsen M. S. Kousholt A. N.. (2011). SET8 is degraded via PCNA-coupled CRL4(CDT2) ubiquitylation in S phase and after UV irradiation. J. Cell Biol. 192, 43–54. 10.1083/jcb.201009076 21220508 PMC3019552

[B84] Kan Z. Jaiswal B. S. Stinson J. Janakiraman V. Bhatt D. Stern H. M.. (2010). Diverse somatic mutation patterns and pathway alterations in human cancers. Nature 466, 869–873. 10.1038/nature09208 20668451

[B85] Kanemori Y. Uto K. Sagata N. (2005). Beta-TrCP recognizes a previously undescribed nonphosphorylated destruction motif in Cdc25A and Cdc25B phosphatases. Proc. Natl. Acad. Sci. U.S.A. 102, 6279–6284. 10.1073/pnas.0501873102 15845771 PMC1083676

[B86] Kapetanaki M. G. Guerrero-Santoro J. Bisi D. C. Hsieh C. L. Rapić-Otrin V. Levine A. S. (2006). The DDB1-CUL4ADDB2 ubiquitin ligase is deficient in xeroderma pigmentosum group E and targets histone H2A at UV-damaged DNA sites. Proc. Natl. Acad. Sci. U.S.A. 103, 2588–2593. 10.1073/pnas.0511160103 16473935 PMC1413840

[B87] Kaur M. Khan M. M. Kar A. Sharma A. Saxena S. (2012). CRL4-DDB1-VPRBP ubiquitin ligase mediates the stress triggered proteolysis of Mcm10. Nucleic Acids Res. 40, 7332–7346. 10.1093/nar/gks366 22570418 PMC3424545

[B88] Kerzendorfer C. Whibley A. Carpenter G. Outwin E. Chiang S. C. Turner G.. (2010). Mutations in Cullin 4B result in a human syndrome associated with increased camptothecin-induced topoisomerase I-dependent DNA breaks. Hum. Mol. Genet. 19, 1324–1334. 10.1093/hmg/ddq008 20064923 PMC2838540

[B89] Kim S. S. Shago M. Kaustov L. Boutros P. C. Clendening J. W. Sheng Y.. (2007). CUL7 is a novel antiapoptotic oncogene. Cancer Res. 67, 9616–9622. 10.1158/0008-5472.CAN-07-0644 17942889

[B90] Kim Y. Kipreos E. T. (2007). Cdt1 degradation to prevent DNA re-replication: conserved and non-conserved pathways. Cell Div. 2:18. 10.1186/1747-1028-2-18 17565698 PMC1913051

[B91] Kim Y. Starostina N. G. Kipreos E. T. (2008). The CRL4Cdt2 ubiquitin ligase targets the degradation of p21Cip1 to control replication licensing. Genes Dev. 22, 2507–2519. 10.1101/gad.1703708 18794348 PMC2546690

[B92] Kitagawa K. Kitagawa M. (2016). The SCF-type E3 ubiquitin ligases as cancer targets. Curr. Cancer Drug Targets 16, 119–129. 10.2174/1568009616666151112122231 26560120

[B93] Knuutila S. Aalto Y. Autio K. Björkqvist A. M. El-Rifai W. Hemmer S.. (1999). DNA copy number losses in human neoplasms. Am. J. Pathol. 155, 683–694. 10.1016/S0002-9440(10)65166-8 10487825 PMC1866903

[B94] Koepp D. M. Kile A. C. Swaminathan S. Rodriguez-Rivera V. (2006). The F-box protein Dia2 regulates DNA replication. Mol. Biol. Cell 17, 1540–1548. 10.1091/mbc.E05-09-0884 16421250 PMC1415285

[B95] Krönke J. Fink E. C. Hollenbach P. W. MacBeth K. J. Hurst S. N. Udeshi N. D.. (2015). Lenalidomide induces ubiquitination and degradation of CK1alpha in del(5q) MDS. Nature 523, 183–188. 10.1038/nature14610 26131937 PMC4853910

[B96] Krönke J. Hurst S. N. Ebert B. L. (2014). Lenalidomide induces degradation of IKZF1 and IKZF3. Oncoimmunology 3:e941742. 10.4161/21624011.2014.941742 25610725 PMC4292522

[B97] Kuang Z. Lewis R. S. Curtis J. M. Zhan Y. Saunders B. M. Babon J. J.. (2010). The SPRY domain-containing SOCS box protein SPSB2 targets iNOS for proteasomal degradation. J. Cell Biol. 190, 129–141. 10.1083/jcb.200912087 20603330 PMC2911665

[B98] Kuchay S. Duan S. Schenkein E. Peschiaroli A. Saraf A. Florens L.. (2013). FBXL2- and PTPL1-mediated degradation of p110-free p85beta regulatory subunit controls the PI(3)K signalling cascade. Nat. Cell Biol. 15, 472–480. 10.1038/ncb2731 23604317 PMC3865866

[B99] Kudo Y. Guardavaccaro D. Santamaria P. G. Koyama-Nasu R. Latres E. Bronson R.. (2004). Role of F-box protein betaTrcp1 in mammary gland development and tumorigenesis. Mol. Cell. Biol. 24, 8184–8194. 10.1128/MCB.24.18.8184-8194.2004 15340078 PMC515055

[B100] Kuznetsova A. V. Meller J. Schnell P. O. Nash J. A. Ignacak M. L. Sanchez Y.. (2003). von Hippel-Lindau protein binds hyperphosphorylated large subunit of RNA polymerase II through a proline hydroxylation motif and targets it for ubiquitination. Proc. Natl. Acad. Sci. U.S.A. 100, 2706–2711. 10.1073/pnas.0436037100 12604794 PMC151405

[B101] Lampert F. Brodersen M. M. Peter M. (2017). Guard the guardian: a CRL4 ligase stands watch over histone production. Nucleus 8, 134–143. 10.1080/19491034.2016.1276143 28072566 PMC5403139

[B102] Le Gallo M. O'Hara A. J. Rudd M. L. Urick M. E. Hansen N. F. O'Neil N. J.. (2012). Exome sequencing of serous endometrial tumors identifies recurrent somatic mutations in chromatin-remodeling and ubiquitin ligase complex genes. Nat. Genet. 44, 1310–1315. 10.1038/ng.2455 23104009 PMC3515204

[B103] Lee Y. R. Yuan W. C. Ho H. C. Chen C. H. Shih H. M. Chen R. H. (2010). The Cullin 3 substrate adaptor KLHL20 mediates DAPK ubiquitination to control interferon responses. EMBO J. 29, 1748–1761. 10.1038/emboj.2010.62 20389280 PMC2876967

[B104] Li C. Ao J. Fu J. Lee D. F. Xu J. Lonard D.. (2011). Tumor-suppressor role for the SPOP ubiquitin ligase in signal-dependent proteolysis of the oncogenic co-activator SRC-3/AIB1. Oncogene 30, 4350–4364. 10.1038/onc.2011.151 21577200 PMC3158261

[B105] Li X. S. Trojer P. Matsumura T. Treisman J. E. Tanese N. (2010). Mammalian SWI/SNF-A Subunit BAF250/ARID1 Is an E3 ubiquitin ligase that targets histone H2B. Mol. Cell. Biol. 30, 1673–1688. 10.1128/MCB.00540-09 20086098 PMC2838063

[B106] Li X. Zhao Q. Liao R. Sun P. Wu X. (2003). The SCF(Skp2) ubiquitin ligase complex interacts with the human replication licensing factor Cdt1 and regulates Cdt1 degradation. J. Biol. Chem. 278, 30854–30858. 10.1074/jbc.C300251200 12840033

[B107] Li Y. Wang X. (2017). The role of cullin4B in human cancers. Exp. Hematol. Oncol. 6:17. 10.1186/s40164-017-0077-2 28630798 PMC5471934

[B108] Li Z. Xiong Y. (2017). Cytoplasmic E3 ubiquitin ligase CUL9 controls cell proliferation, senescence, apoptosis and genome integrity through p53. Oncogene 36, 5212–5218. 10.1038/onc.2017.141 28481879 PMC5589481

[B109] Li Z. Pei X. H. Yan J. Yan F. Cappell K. M. Whitehurst A. W.. (2014). CUL9 mediates the functions of the 3M complex and ubiquitylates survivin to maintain genome integrity. Mol. Cell 54, 805–819. 10.1016/j.molcel.2014.03.046 24793696 PMC4172312

[B110] Limón-Mortés M. C. Mora-Santos M. Espina A. Pintor-Toro J. A. Lopez-Roman A. Tortolero M.. (2008). UV-induced degradation of securin is mediated by SKP1-CUL1-beta TrCP E3 ubiquitin ligase. J. Cell Sci. 121, 1825–1831. 10.1242/jcs.020552 18460583

[B111] Lin P. Fu J. Zhao B. Lin F. Zou H. Liu L. Y.. (2011). Fbxw8 is involved in the proliferation of human choriocarcinoma JEG-3 cells. Mol. Biol. Rep. 38, 1741–1747. 10.1007/s11033-010-0288-7 20878477

[B112] Litterman N. Ikeuchi Y. Gallardo G. O'Connell B. C. Sowa M. E. Gygi S. P.. (2011). An OBSL1-Cul7Fbxw8 ubiquitin ligase signaling mechanism regulates Golgi morphology and dendrite patterning. PLoS Biol. 9:e1001060. 10.1371/journal.pbio.1001060 21572988 PMC3091842

[B113] Liu S. Nheu T. Luwor R. Nicholson S. E. Zhu H. J. (2015). SPSB1, a novel negative regulator of the transforming growth factor-beta signaling pathway targeting the Type II receptor. J. Biol. Chem. 290, 17894–17908. 10.1074/jbc.M114.607184 26032413 PMC4505038

[B114] Lo Y. H. Ho P. C. Wang S. C. (2012). Epidermal growth factor receptor protects proliferating cell nuclear antigen from Cullin 4A protein-mediated proteolysis. J. Biol. Chem. 287, 27148–27157. 10.1074/jbc.M112.388843 22692198 PMC3411057

[B115] Ma C. Q. Qi Y. Shao L. P. Liu M. Li X. Tang H. (2013). Downregulation of miR-7 Upregulates Cullin 5 (CUL5) to facilitate G1/S transition in human Hepatocellular carcinoma cells. IUBMB Life 65, 1026–1034. 10.1002/iub.1231 24339204

[B116] Machado-Oliveira G. Guerreiro E. Matias A. C. Facucho-Oliveira J. Pacheco-Leyva I. Bragança J. (2015). FBXL5 modulates HIF-1alpha transcriptional activity by degradation of CITED2. Arch. Biochem. Biophys. 576, 61–72. 10.1016/j.abb.2015.04.012 25956243

[B117] Maculins T. Nkosi P. J. Nishikawa H. Labib K. (2015). Tethering of SCF(Dia2) to the replisome promotes efficient ubiquitylation and disassembly of the CMG helicase. Curr. Biol. 25, 2254–2259. 10.1016/j.cub.2015.07.012 26255844 PMC4562905

[B118] Mallampalli R. K. Kaercher L. Snavely C. Pulijala R. Chen B. B. Coon T.. (2013). Fbxl12 triggers G1 arrest by mediating degradation of calmodulin kinase I. Cell. Signal. 25, 2047–2059. 10.1016/j.cellsig.2013.05.012 23707388 PMC3905573

[B119] Maniaci C. Hughes S. J. Testa A. Chen W. Lamont D. J. Rocha S.. (2017). Homo-PROTACs: bivalent small-molecule dimerizers of the VHL E3 ubiquitin ligase to induce self-degradation. Nat. Commun. 8:830. 10.1038/s41467-017-00954-1 29018234 PMC5635026

[B120] Margottin-Goguet F. Hsu J. Y. Loktev A. Hsieh H. M. Reimann J. D. Jackson P. K. (2003). Prophase destruction of Emi1 by the SCF(betaTrCP/Slimb) ubiquitin ligase activates the anaphase promoting complex to allow progression beyond prometaphase. Dev. Cell 4, 813–826. 10.1016/S1534-5807(03)00153-9 12791267

[B121] Masoud G. N. Li W. (2015). HIF-1alpha pathway: role, regulation and intervention for cancer therapy. Acta Pharm. Sin. B 5, 378–389. 10.1016/j.apsb.2015.05.007 26579469 PMC4629436

[B122] Maynard M. A. Ohh M. (2004). von Hippel-Lindau tumor suppressor protein and hypoxia-inducible factor in kidney cancer. Am. J. Nephrol. 24, 1–13. 10.1159/000075346 14654728

[B123] McMahon M. Itoh K. Yamamoto M. Hayes J. D. (2003). Keap1-dependent proteasomal degradation of transcription factor Nrf2 contributes to the negative regulation of antioxidant response element-driven gene expression. J. Biol. Chem. 278, 21592–21600. 10.1074/jbc.M300931200 12682069

[B124] Men X. Wang L. Yu W. Ju Y. (2014). Cullin7 is required for lung cancer cell proliferation and is overexpressed in lung cancer. Oncol. Res. 22, 123–128. 10.3727/096504014X14198596979742 25706399 PMC7838442

[B125] Merlet J. Burger J. Tavernier N. Richaudeau B. Gomes J. E. Pintard L. (2010). The CRL2LRR-1 ubiquitin ligase regulates cell cycle progression during *C. elegans* development. Development 137, 3857–3866. 10.1242/dev.054866 20978077 PMC3188596

[B126] Mulvaney K. M. Matson J. P. Siesser P. F. Tamir T. Y. Goldfarb D. Jacobs T. M.. (2016). Identification and characterization of MCM3 as a kelch-like ECH-associated Protein 1 (KEAP1) substrate. J. Biol. Chem. 291, 23719–23733. 10.1074/jbc.M116.729418 27621311 PMC5095425

[B127] Muniz J. R. Guo K. Kershaw N. J. Ayinampudi V. von Delft F. Babon J. J.. (2013). Molecular architecture of the ankyrin SOCS box family of Cul5-dependent E3 ubiquitin ligases. J. Mol. Biol. 425, 3166–3177. 10.1016/j.jmb.2013.06.015 23806657 PMC3779351

[B128] Nagel S. Pommerenke C. Meyer C. Kaufmann M. MacLeod R. A. F. Drexler H. G. (2017). Identification of a tumor suppressor network in T-cell leukemia. Leuk. Lymphoma 58, 2196–2207. 10.1080/10428194.2017.1283029 28142295

[B129] Nakayama K. I. Hatakeyama S. Nakayama K. (2001). Regulation of the cell cycle at the G1-S transition by proteolysis of cyclin E and p27Kip1. Biochem. Biophys. Res. Commun. 282, 853–860. 10.1006/bbrc.2001.4627 11352628

[B130] Nakayama K. Nagahama H. Minamishima Y. A. Matsumoto M. Nakamichi I. Kitagawa K.. (2000). Targeted disruption of Skp2 results in accumulation of cyclin E and p27(Kip1), polyploidy and centrosome overduplication. EMBO J. 19, 2069–2081. 10.1093/emboj/19.9.2069 10790373 PMC305685

[B131] Nakayama K. Nagahama H. Minamishima Y. A. Miyake S. Ishida N. Hatakeyama S.. (2004). Skp2-mediated degradation of p27 regulates progression into mitosis. Dev. Cell 6, 661–672. 10.1016/S1534-5807(04)00131-5 15130491

[B132] Nishitani H. Shiomi Y. Iida H. Michishita M. Takami T. Tsurimoto T. (2008). CDK inhibitor p21 is degraded by a proliferating cell nuclear antigen-coupled Cul4-DDB1Cdt2 pathway during S phase and after UV irradiation. J. Biol. Chem. 283, 29045–29052. 10.1074/jbc.M806045200 18703516 PMC2662008

[B133] Nishiya T. Matsumoto K. Maekawa S. Kajita E. Horinouchi T. Fujimuro M.. (2011). Regulation of inducible nitric-oxide synthase by the SPRY domain- and SOCS box-containing proteins. J. Biol. Chem. 286, 9009–9019. 10.1074/jbc.M110.190678 21199876 PMC3058989

[B134] Ohh M. Park C. W. Ivan M. Hoffman M. A. Kim T. Y. Huang L. E.. (2000). Ubiquitination of hypoxia-inducible factor requires direct binding to the beta-domain of the von Hippel-Lindau protein. Nat. Cell Biol. 2, 423–427. 10.1038/35017054 10878807

[B135] Okabe H. Lee S. H. Phuchareon J. Albertson D. G. McCormick F. Tetsu O. (2006). A critical role for FBXW8 and MAPK in cyclin D1 degradation and cancer cell proliferation. PLoS ONE 1:e128. 10.1371/journal.pone.0000128 17205132 PMC1762433

[B136] Okumura F. Joo-Okumura A. Nakatsukasa K. Kamura T. (2016). The role of cullin 5-containing ubiquitin ligases. Cell Div. 11:1. 10.1186/s13008-016-0016-3 27030794 PMC4812663

[B137] Oladghaffari M. Islamian J. P. Baradaran B. Monfared A. S. (2016). MLN4924 therapy as a novel approach in cancer treatment modalities. J. Chemother. 28, 74–82. 10.1179/1973947815Y.0000000066 26292710

[B138] Oladghaffari M. Monfared A. Farajollahi A. Baradaran B. Mohammadi M. Shanehbandi D.. (2017). MLN4924 and 2DG combined treatment enhances the efficiency of radiotherapy in breast cancer cells. Int. J. Radiat. Biol. 93, 590–599. 10.1080/09553002.2017.1294272 28291374

[B139] Paradis V. Albuquerque M. Mebarki M. Hernandez L. Zalinski S. Quentin S.. (2013). Cullin7: a new gene involved in liver carcinogenesis related to metabolic syndrome. Gut 62, 911–919. 10.1136/gutjnl-2012-302091 22942238

[B140] Park S. W. Chung N. G. Hur S. Y. Kim H. S. Yoo N. J. Lee S. H. (2009). Mutational analysis of hypoxia-related genes HIF1alpha and CUL2 in common human cancers. APMIS 117, 880–885. 10.1111/j.1600-0463.2009.02550.x 20078552

[B141] Parker M. W. Botchan M. R. Berger J. M. (2017). Mechanisms and regulation of DNA replication initiation in eukaryotes. Crit. Rev. Biochem. Mol. Biol. 52, 107–144. 10.1080/10409238.2016.1274717 28094588 PMC5545932

[B142] Pateras I. S. Apostolopoulou K. Koutsami M. Evangelou K. Tsantoulis P. Liloglou T.. (2006). Downregulation of the KIP family members p27(KIP1) and p57(KIP2) by SKP2 and the role of methylation in p57(KIP2) inactivation in nonsmall cell lung cancer. Int. J. Cancer 119, 2546–2556. 10.1002/ijc.22214 16988944

[B143] Pause A. Lee S. Worrell R. A. Chen D. Y. Burgess W. H. Linehan W. M.. (1997). The von Hippel-Lindau tumor-suppressor gene product forms a stable complex with human CUL-2, a member of the Cdc53 family of proteins. Proc. Natl. Acad. Sci. U.S.A. 94, 2156–2161. 10.1073/pnas.94.6.2156 9122164 PMC20057

[B144] Pei X. H. Bai F. Li Z. J. Smith M. D. Whitewolf G. Jin R.. (2011). Cytoplasmic CUL9/PARC ubiquitin ligase is a tumor suppressor and promotes p53-Dependent apoptosis. Cancer Res. 71, 2969–2977. 10.1158/0008-5472.CAN-10-4300 21487039 PMC3088989

[B145] Peng Z. M. Liao Z. P. Matsumoto Y. Yang A. Tomkinson A. E. (2016). Human DNA Ligase I interacts with and is targeted for degradation by the DCAF7 specificity factor of the Cul4-DDB1 ubiquitin ligase complex. J. Biol. Chem. 291, 21893–21902. 10.1074/jbc.M116.746198 27573245 PMC5063974

[B146] Peschiaroli A. Dorrello N. V. Guardavaccaro D. Venere M. Halazonetis T. Sherman N. E.. (2006). SCFbetaTrCP-mediated degradation of claspin regulates recovery from the DNA replication checkpoint response. Mol. Cell 23, 319–329. 10.1016/j.molcel.2006.06.013 16885022

[B147] Petzold G. Fischer E. S. Thoma N. H. (2016). Structural basis of lenalidomide-induced CK1alpha degradation by the CRL4(CRBN) ubiquitin ligase. Nature 532, 127–130. 10.1038/nature16979 26909574

[B148] Pintard L. Willems A. Peter M. (2004). Cullin-based ubiquitin ligases: Cul3-BTB complexes join the family. EMBO J. 23, 1681–1687. 10.1038/sj.emboj.7600186 15071497 PMC394240

[B149] Pozo P. N. Cook J. G. (2016). Regulation and function of Cdt1; a key factor in cell proliferation and genome stability. Genes 8:2. 10.3390/genes8010002 28025526 PMC5294997

[B150] Qiao S. S. Guo W. W. Liao L. J. Wang L. Wang Z. Zhang R.. (2015). DDB2 is involved in ubiquitination and degradation of PAQR3 and regulates tumorigenesis of gastric cancer cells. Biochem. J. 469, 469–480. 10.1042/BJ20150253 26205499

[B151] Ray D. Osmundson E. C. Kiyokawa H. (2006). Constitutive and UV-induced fibronectin degradation is a ubiquitination-dependent process controlled by beta-TrCP. J. Biol. Chem. 281, 23060–23065. 10.1074/jbc.M604311200 16757476

[B152] Ren W. G. Sun Z. Q. Zeng Q. L. Han S. Zhang Q. L. Jiang L. B. (2016). Aberrant expression of CUL4A is associated with IL-6/STAT3 activation in colorectal cancer progression. Arch. Med. Res. 47, 214–222. 10.1016/j.arcmed.2016.07.001 27418574

[B153] Rowbotham D. A. Enfield K. S. Martinez V. D. Thu K. L. Vucic E. A. Stewart G. L.. (2014). Multiple components of the VHL tumor suppressor complex are frequently affected by DNA copy number loss in Pheochromocytoma. Int. J. Endocrinol. 2014:546347. 10.1155/2014/546347 25298778 PMC4178909

[B154] Sakaue T. Sakakibara I. Uesugi T. Fujisaki A. Nakashiro K. I. Hamakawa H. (2017). The CUL3-SPOP-DAXX axis is a novel regulator of VEGFR2 expression in vascular endothelial cells. Sci. Rep. 7:42845 10.1038/srep42845 28216678 PMC5317005

[B155] Sarantopoulos J. Shapiro G. I. Cohen R. B. Clark J. W. Kauh J. S. Weiss G. J.. (2016). Phase I study of the investigational NEDD8-Activating enzyme inhibitor pevonedistat (TAK-924/MLN4924) in patients with advanced solid tumors. Clin. Cancer Res. 22, 847–857. 10.1158/1078-0432.CCR-15-1338 26423795

[B156] Sarikas A. Hartmann T. Pan Z. Q. (2011). The cullin protein family. Genome Biol. 12:220. 10.1186/gb-2011-12-4-220 21554755 PMC3218854

[B157] Sarikas A. Xu X. Field L. J. Pan Z. Q. (2008). The cullin7 E3 ubiquitin ligase: a novel player in growth control. Cell Cycle 7, 3154–3161. 10.4161/cc.7.20.6922 18927510 PMC2637179

[B158] Savvidis C. Koutsilieris M. (2012). Circadian rhythm disruption in cancer biology. Mol. Med. 18, 1249–1260. 10.2119/molmed.2012.00077 22811066 PMC3521792

[B159] Seipel K. Marques M. T. Bozzini M. A. Meinken C. Mueller B. U. Pabst T. (2016). Inactivation of the p53-KLF4-CEBPA axis in acute Myeloid Leukemia. Clin. Cancer Res. 22, 746–756. 10.1158/1078-0432.CCR-15-1054 26408402

[B160] Shah J. J. Jakubowiak A. J. O'Connor O. A. Orlowski R. Z. Harvey R. D. Smith M. R. (2016). Phase I Study of the novel investigational NEDD8-activating enzyme inhibitor pevonedistat (MLN4924) in patients with relapsed/refractory Multiple Myeloma or Lymphoma. Clin. Cancer Res. 22, 34–43. 10.1158/1078-0432.CCR-15-1237 26561559 PMC5694347

[B161] Sharma P. Nag A. (2014). CUL4A ubiquitin ligase: a promising drug target for cancer and other human diseases. Open Biol. 4:130217. 10.1098/rsob.130217 24522884 PMC3938054

[B162] Shen Z. Prasanth S. G. (2012). Orc2 protects ORCA from ubiquitin-mediated degradation. Cell Cycle 11, 3578–3589. 10.4161/cc.21870 22935713 PMC3478309

[B163] Shi Y. Q. Liao S. Y. Zhuang X. J. Han C. S. (2011). Mouse Fem1b interacts with and induces ubiquitin-mediated degradation of Ankrd37. Gene 485, 153–159. 10.1016/j.gene.2011.06.025 21723927

[B164] Shibutani S. T. de la Cruz A. F. Tran V. Turbyfill W. J. III. Reis T. Edgar B. A.. (2008). Intrinsic negative cell cycle regulation provided by PIP box- and Cul4Cdt2-mediated destruction of E2f1 during S phase. Dev. Cell 15, 890–900. 10.1016/j.devcel.2008.10.003 19081076 PMC2644461

[B165] Shim E. H. Johnson L. Noh H. L. Kim Y. J. Sun H. Zeiss C.. (2003). Expression of the F-box protein SKP2 induces hyperplasia, dysplasia, and low-grade carcinoma in the mouse prostate. Cancer Res. 63, 1583–1588. 12670908

[B166] Siepka S. M. Yoo S. H. Park J. Song W. M. Kumar V. Hu Y. N.. (2007). Circadian mutant overtime reveals F-box protein FBXL3 regulation of cryptochrome and period gene expression. Cell 129, 1011–1023. 10.1016/j.cell.2007.04.030 17462724 PMC3762874

[B167] Sistrunk C. Kim S. H. Wang X. Lee S. H. Kim Y. Macias E.. (2013). Skp2 deficiency inhibits chemical skin tumorigenesis independent of p27(Kip1) accumulation. Am. J. Pathol. 182, 1854–1864. 10.1016/j.ajpath.2013.01.016 23474082 PMC3644727

[B168] Skaar J. R. Pagan J. K. Pagano M. (2013). Mechanisms and function of substrate recruitment by F-box proteins. Nat. Rev. Mol. Cell Biol. 14, 369–381. 10.1038/nrm3582 23657496 PMC3827686

[B169] Slenn T. J. Morris B. Havens C. G. Freeman R. M. Jr. Takahashi T. S. Walter J. C. (2014). Thymine DNA glycosylase is a CRL4Cdt2 substrate. J. Biol. Chem. 289, 23043–23055. 10.1074/jbc.M114.574194 24947512 PMC4132803

[B170] Song J. Zhang J. Shao J. (2015). Knockdown of CUL4A inhibits invasion and induces apoptosis in osteosarcoma cells. Int. J. Immunopathol. Pharmacol. 28, 263–269. 10.1177/0394632015586656 26055549

[B171] Sonneville R. Moreno S. P. Knebel A. Johnson C. Hastie C. J. Gartner A.. (2017). CUL-2LRR-1 and UBXN-3 drive replisome disassembly during DNA replication termination and mitosis. Nat. Cell Biol. 19, 468–479. 10.1038/ncb3500 28368371 PMC5410169

[B172] Soucy T. A. Dick L. R. Smith P. G. Milhollen M. A. Brownell J. E. (2010). The NEDD8 conjugation pathway and its relevance in cancer biology and therapy. Genes Cancer 1, 708–716. 10.1177/1947601910382898 21779466 PMC3092238

[B173] Soucy T. A. Smith P. G. Milhollen M. A. Berger A. J. Gavin J. M. Adhikari S.. (2009). An inhibitor of NEDD8-activating enzyme as a new approach to treat cancer. Nature 458, 732–736. 10.1038/nature07884 19360080

[B174] Starostina N. G. Simpliciano J. M. McGuirk M. A. Kipreos E. T. (2010). CRL2(LRR-1) targets a CDK inhibitor for cell cycle control in *C. elegans* and actin-based motility regulation in human cells. Dev. Cell 19, 753–764. 10.1016/j.devcel.2010.10.013 21074724 PMC3053091

[B175] Stogios P. J. Downs G. S. Jauhal J. J. Nandra S. K. Prive G. G. (2005). Sequence and structural analysis of BTB domain proteins. Genome Biol. 6:R82. 10.1186/gb-2005-6-10-r82 16207353 PMC1257465

[B176] Su Y. Ni Z. Wang G. Cui J. Wei C. Wang J.. (2012). Aberrant expression of microRNAs in gastric cancer and biological significance of miR-574-3p. Int. Immunopharmacol. 13, 468–475. 10.1016/j.intimp.2012.05.016 22683180 PMC3389336

[B177] Swords R. T. Erba H. P. DeAngelo D. J. Bixby D. L. Altman J. K. Maris M.. (2015). Pevonedistat (MLN4924), a First-in-Class NEDD8-activating enzyme inhibitor, in patients with acute myeloid leukaemia and myelodysplastic syndromes: a phase 1 study. Br. J. Haematol. 169, 534–543. 10.1111/bjh.13323 25733005

[B178] Takeishi S. Nakayama K. I. (2014). Role of Fbxw7 in the maintenance of normal stem cells and cancer-initiating cells. Br. J. Cancer 111, 1054–1059. 10.1038/bjc.2014.259 24853181 PMC4453837

[B179] Tan M. J. Gallegos J. R. Gu Q. Y. Huang Y. H. Li J. Jin Y. T.. (2006). SAG/ROC-SCF beta-TrCP E3 ubiquitin ligase promotes pro-caspase-3 degradation as a mechanism of apoptosis protection. Neoplasia 8, 1042–1054. 10.1593/neo.06568 17217622 PMC1783718

[B180] Tsukada Y. Fang J. Erdjument-Bromage H. Warren M. E. Borchers C. H. Tempst P.. (2006). Histone demethylation by a family of JmjC domain-containing proteins. Nature 439, 811–816. 10.1038/nature04433 16362057

[B181] Tu Y. Liu H. Zhu X. Shen H. Ma X. Wang F.. (2017). Ataxin-3 promotes genome integrity by stabilizing Chk1. Nucleic Acids Res. 45, 4532–4549. 10.1093/nar/gkx095 28180282 PMC5416811

[B182] Tzatsos A. Paskaleva P. Ferrari F. Deshpande V. Stoykova S. Contino G.. (2013). KDM2B promotes pancreatic cancer via Polycomb-dependent and -independent transcriptional programs. J. Clin. Invest. 123, 727–739. 10.1172/JCI64535 23321669 PMC3561797

[B183] Uchida S. Watanabe N. Kudo Y. Yoshioka K. Matsunaga T. Ishizaka Y.. (2011). SCFbeta(TrCP) mediates stress-activated MAPK-induced Cdc25B degradation. J. Cell. Sci. 124(Pt 16), 2816–2825. 10.1242/jcs.083931 21807946

[B184] Ueda T. Nagamachi A. Takubo K. Yamasaki N. Matsui H. Kanai A.. (2015). Fbxl10 overexpression in murine hematopoietic stem cells induces leukemia involving metabolic activation and upregulation of Nsg2. Blood 125, 3437–3446. 10.1182/blood-2014-03-562694 25872778 PMC4447860

[B185] Vaid M. Prasad R. Sun Q. Katiyar S. K. (2011). Silymarin targets beta-catenin signaling in blocking migration/invasion of human melanoma cells. PLoS ONE 6:e23000. 10.1371/journal.pone.0023000 21829575 PMC3145779

[B186] Vanderdys V. Allak A. Guessous F. Benamar M. Read P. W. Jameson M. J.. (2017). The Neddylation Inhibitor Pevonedistat (MLN4924) suppresses and radiosensitizes head and neck squamous carcinoma cells and tumors. Mol. Cancer Ther. 17, 368–380. 10.1158/1535-7163 28838998 PMC5805645

[B187] Vaupel P. Mayer A. (2007). Hypoxia in cancer: significance and impact on clinical outcome. Cancer Metastasis Rev. 26, 225–239. 10.1007/s10555-007-9055-1 17440684

[B188] Viñas-Castells R. Beltran M. Valls G. Gómez I. García J. M. Montserrat-Sentís B.. (2010). The hypoxia-controlled FBXL14 ubiquitin ligase targets SNAIL1 for proteasome degradation. J. Biol. Chem. 285, 3794–3805. 10.1074/jbc.M109.065995 19955572 PMC2823521

[B189] Walter D. Hoffmann S. Komseli E. S. Rappsilber J. Gorgoulis V. Sorensen C. S. (2016). SCF(Cyclin F)-dependent degradation of CDC6 suppresses DNA re-replication. Nat. Commun. 7:10530. 10.1038/ncomms10530 26818844 PMC4738361

[B190] Wan J. F. Zhu J. Li G. C. Zhang Z. (2016). Radiosensitization of human colorectal cancer cells by MLN4924: an inhibitor of NEDD8-Activating enzyme. Technol. Cancer Res. Treat. 15, 527–534. 10.1177/1533034615588197 26082455

[B191] Wang H. Chen Y. Lin P. Li L. Zhou G. S. Liu G. C.. (2014). The CUL7/F-box and WD repeat domain containing 8 (CUL7/Fbxw8) ubiquitin ligase promotes degradation of hematopoietic Progenitor Kinase 1. J. Biol. Chem. 289, 4009–4017. 10.1074/jbc.M113.520106 24362026 PMC3924268

[B192] Wang X. F. Zhang W. J. Yan Z. Liang Y. P. Li L. H. Yu X. L.. (2016). Radiosensitization by the investigational NEDD8-activating enzyme inhibitor MLN4924 (pevonedistat) in hormone-resistant prostate cancer cells. Oncotarget 7, 38380–38391. 10.18632/oncotarget.9526 27224919 PMC5122397

[B193] Wang X. Arceci A. Bird K. Mills C. A. Choudhury R. Kernan J. L.. (2017). VprBP/DCAF1 regulates the degradation and nonproteolytic activation of the cell cycle transcription factor FoxM1. Mol. Cell. Biol. 37:e00609-16. 10.1128/MCB.00609-16 28416635 PMC5472828

[B194] Wang Z. Dai X. Zhong J. Inuzuka H. Wan L. Li X.. (2015). SCF(beta-TRCP) promotes cell growth by targeting PR-Set7/Set8 for degradation. Nat. Commun. 6:10185. 10.1038/ncomms10185 26666832 PMC4682171

[B195] Watanabe N. Arai H. Nishihara Y. Taniguchi M. Watanabe N. Hunter T.. (2004). M-phase kinases induce phospho-dependent ubiquitination of somatic Wee1 by SCFbeta-TrCP. Proc. Natl. Acad. Sci. U.S.A. 101, 4419–4424. 10.1073/pnas.0307700101 15070733 PMC384762

[B196] Wei D. P. Li H. Yu J. Sebolt J. T. Zhao L. Lawrence T. S.. (2012). Radiosensitization of human pancreatic cancer cells by MLN4924, an investigational NEDD8-Activating enzyme inhibitor. Cancer Res. 72, 282–293. 10.1158/0008-5472.CAN-11-2866 22072567 PMC3251739

[B197] Wei S. Yang H. C. Chuang H. C. Yang J. Kulp S. K. Lu P. J.. (2008). A novel mechanism by which thiazolidinediones facilitate the proteasomal degradation of cyclin D1 in cancer cells. J. Biol. Chem. 283, 26759–26770. 10.1074/jbc.M802160200 18650423 PMC2546561

[B198] Wertz I. E. Kusam S. Lam C. Okamoto T. Sandoval W. Anderson D. J.. (2011). Sensitivity to antitubulin chemotherapeutics is regulated by MCL1 and FBW7. Nature 471, 110–114. 10.1038/nature09779 21368834

[B199] Wu W. D. Ding H. H. Cao J. Zhang W. H. (2015). FBXL5 inhibits metastasis of gastric cancer through suppressing snail1. Cell. Physiol. Biochem. 35, 1764–1772. 10.1159/000373988 25832584

[B200] Wu X. D. Johansen J. V. Helin K. (2013). Fbxl10/kdm2b recruits polycomb repressive complex 1 to CpG islands and regulates H2A ubiquitylation. Mol. Cell 49, 1134–1146. 10.1016/j.molcel.2013.01.016 23395003

[B201] Xiao J. Zhang T. Xu D. Wang H. Cai Y. Jin T.. (2015). FBXL20-mediated Vps34 ubiquitination as a p53 controlled checkpoint in regulating autophagy and receptor degradation. Genes Dev. 29, 184–196. 10.1101/gad.252528.114 25593308 PMC4298137

[B202] Xie P. Yang J. P. Cao Y. Peng L. X. Zheng L. S. Sun R.. (2017). Promoting tumorigenesis in nasopharyngeal carcinoma, NEDD8 serves as a potential theranostic target. Cell Death Dis. 8:e2834. 10.1038/cddis.2017.195 28569775 PMC5520881

[B203] Xu G. Bernaudo S. Fu G. Lee D. Y. Yang B. B. Peng C. (2008). Cyclin G2 is degraded through the ubiquitin-proteasome pathway and mediates the antiproliferative effect of activin receptor-like kinase 7. Mol. Biol. Cell 19, 4968–4979. 10.1091/mbc.E08-03-0259 18784254 PMC2575152

[B204] Xu J. Fang Y. Wang X. Wang F. Tian Q. Li Y.. (2016). CUL2 overexpression driven by CUL2/E2F1/miR-424 regulatory loop promotes HPV16 E7 induced cervical carcinogenesis. Oncotarget 7, 31520–31533. 10.18632/oncotarget.9127 27153550 PMC5058775

[B205] Xu J. Li L. Yu G. Ying W. Gao Q. Zhang W.. (2015). The neddylation-cullin 2-RBX1 E3 ligase axis targets tumor suppressor RhoB for degradation in liver cancer. Mol. Cell. Proteomics 14, 499–509. 10.1074/mcp.M114.045211 25540389 PMC4349972

[B206] Xu J. Zhou J. Y. Xu Z. Kho D. H. Zhuang Z. Raz A.. (2014). The role of Cullin3-mediated ubiquitination of the catalytic subunit of PP2A in TRAIL signaling. Cell Cycle 13, 3750–3758. 10.4161/15384101.2014.965068 25551360 PMC4612120

[B207] Xu X. M. Wang X. B. Chen M. M. Liu T. Li Y. X. Jia W. H.. (2012). MicroRNA-19a and−19b regulate cervical carcinoma cell proliferation and invasion by targeting CUL5. Cancer Lett. 322, 148–158. 10.1016/j.canlet.2012.02.038 22561557

[B208] Xu X. Sarikas A. Dias-Santagata D. C. Dolios G. Lafontant P. J. Tsai S. C.. (2008). The CUL7 E3 ubiquitin ligase targets insulin receptor substrate 1 for ubiquitin-dependent degradation. Mol. Cell 30, 403–414. 10.1016/j.molcel.2008.03.009 18498745 PMC2633441

[B209] Yang Y. Liu R. Qiu R. Zheng Y. Huang W. Hu H.. (2015). CRL4B promotes tumorigenesis by coordinating with SUV39H1/HP1/DNMT3A in DNA methylation-based epigenetic silencing. Oncogene 34, 104–118. 10.1038/onc.2013.522 24292684

[B210] Yu Z. K. Gervais J. L. Zhang H. (1998). Human CUL-1 associates with the SKP1/SKP2 complex and regulates p21(CIP1/WAF1) and cyclin D proteins. Proc. Natl. Acad. Sci. U.S.A. 95, 11324–11329. 10.1073/pnas.95.19.11324 9736735 PMC21641

[B211] Yuan W. C. Lee Y. R. Huang S. F. Lin Y. M. Chen T. Y. Chung H. C.. (2011). A Cullin3-KLHL20 ubiquitin Ligase-Dependent pathway targets PML to potentiate HIF-1 signaling and prostate cancer progression. Cancer Cell 20, 214–228. 10.1016/j.ccr.2011.07.008 21840486

[B212] Zaidi I. W. Rabut G. Poveda A. Scheel H. Malmstrom J. Ulrich H.. (2008). Rtt101 and Mms1 in budding yeast form a CUL4(DDB1)-like ubiquitin ligase that promotes replication through damaged DNA. EMBO Rep. 9, 1034–1040. 10.1038/embor.2008.155 18704118 PMC2572122

[B213] Zhang D. H. Yang G. L. Li X. D. Xu C. Ge H. L. (2016). Inhibition of liver carcinoma cell invasion and metastasis by knockdown of Cullin7 *in vitro* and *in vivo*. Oncol. Res. 23, 171–181. 10.3727/096504016X14519995067562 27053346 PMC7838605

[B214] Zhang H. F. Tomida A. Koshimizu R. Ogiso Y. Lei S. Tsuruo T. (2004). Cullin 3 promotes proteasomal degradation of the topoisomerase I-DNA covalent complex. Cancer Res. 64, 1114–1121. 10.1158/0008-5472.CAN-03-2858 14871846

[B215] Zhang S. Zhao H. Darzynkiewicz Z. Zhou P. Zhang Z. Lee E. Y.. (2013). A novel function of CRL4(Cdt2): regulation of the subunit structure of DNA polymerase delta in response to DNA damage and during the S phase. J. Biol. Chem. 288, 29550–29561. 10.1074/jbc.M113.490466 23913683 PMC3795253

[B216] Zhang Y. Huang L. Fu H. Smith O. K. Lin C. M. Utani K.. (2016). A replicator-specific binding protein essential for site-specific initiation of DNA replication in mammalian cells. Nat. Commun. 7:11748. 10.1038/ncomms11748 27272143 PMC4899857

[B217] Zheng N. N. Zhou Q. S. Wang Z. W. Wei W. Y. (2016). Recent advances in SCF ubiquitin ligase complex: clinical implications. Biochim. Biophys. Acta 1866, 12–22. 10.1016/j.bbcan.2016.05.001 27156687 PMC4980229

[B218] Zheng N. Schulman B. A. Song L. Miller J. J. Jeffrey P. D. Wang P.. (2002). Structure of the Cul1-Rbx1-Skp1-F boxSkp2 SCF ubiquitin ligase complex. Nature 416, 703–709. 10.1038/416703a 11961546

[B219] Zhong H. De Marzo A. M. Laughner E. Lim M. Hilton D. A. Zagzag D.. (1999). Overexpression of hypoxia-inducible factor 1alpha in common human cancers and their metastases. Cancer Res. 59, 5830–5835. 10582706

[B220] Zhong W. Feng H. Santiago F. E. Kipreos E. T. (2003). CUL-4 ubiquitin ligase maintains genome stability by restraining DNA-replication licensing. Nature 423, 885–889. 10.1038/nature01747 12815436

[B221] Zhu Z. Wang L. Hao R. Zhao B. Sun L. Ye R. D. (2016). Cutting Edge: a Cullin-5-TRAF6 interaction promotes TRAF6 polyubiquitination and Lipopolysaccharide Signaling. J. Immunol. 197, 21–26. 10.4049/jimmunol.1600447 27233966

[B222] Zhuang M. Calabrese M. F. Liu J. Waddell M. B. Nourse A. Hammel M.. (2009). Structures of SPOP-Substrate complexes: insights into molecular architectures of BTB-Cul3 ubiquitin ligases. Mol. Cell 36, 39–50. 10.1016/j.molcel.2009.09.022 19818708 PMC2847577

[B223] Zimmerman E. S. Schulman B. A. Zheng N. (2010). Structural assembly of cullin-RING ubiquitin ligase complexes. Curr. Opin. Struct. Biol. 20, 714–721. 10.1016/j.sbi.2010.08.010 20880695 PMC3070871

[B224] Zou C. B. Chen Y. Smith R. M. Snavely C. Li J. Coon T. A.. (2013). SCFFbxw15 mediates histone acetyltransferase binding to origin recognition complex (HBO1) ubiquitin-proteasomal degradation to regulate cell proliferation. J. Biol. Chem. 288, 6306–6316. 10.1074/jbc.M112.426882 23319590 PMC3585065

[B225] Zou Y. Mi J. Wang W. Lu J. Zhao W. Liu Z.. (2013). CUL4B promotes replication licensing by up-regulating the CDK2-CDC6 cascade. J. Cell Biol. 200, 743–756. 10.1083/jcb.201206065 23479742 PMC3601365

